# Hemorrhage Risk Associated with Anticoagulant and Antiplatelet Drug Combinations: Insights from the USFDA Adverse Event Reporting System

**DOI:** 10.3390/jcm14176262

**Published:** 2025-09-04

**Authors:** Kannan Sridharan, Gowri Sivaramakrishnan

**Affiliations:** 1 Department of Pharmacology & Therapeutics, College of Medicine & Health Sciences, Arabian Gulf University, Manama 26671, Bahrain; 2 Bahrain Defence Force Royal Medical Services, Riffa 28743, Bahrain; gowri.sivaramakrishnan@gmail.com

**Keywords:** warfarin, apixaban, aspirin, clopidogrel, dabigatran, dipyridamole, cilostazol

## Abstract

**Background:** While anticoagulant and antiplatelet therapies are commonly combined in clinical settings, this combination increases the risk of hemorrhage. However, comparative data on the bleeding risks of different drug combinations remain limited. This study assesses hemorrhage risk associated with various anticoagulant–antiplatelet combinations using data from the USFDA Adverse Event Reporting System (AERS). **Methods:** Hemorrhage-related reports were extracted from the AERS database (March 2004–June 2024). Anticoagulants analyzed included warfarin, rivaroxaban, dabigatran, apixaban, edoxaban, betrixaban, and acenocoumarol; antiplatelets included aspirin, clopidogrel, ticagrelor, cilostazol, prasugrel, and dipyridamole. Disproportionality analysis using frequentist and Bayesian approaches was conducted to detect hemorrhage signals. **Results:** Out of 160,715 hemorrhage reports, rivaroxaban, warfarin, and apixaban were the most frequently reported anticoagulants, while aspirin and clopidogrel were the top antiplatelets. Apixaban had the lowest reporting odds ratio for hemorrhage. The rivaroxaban-aspirin combination showed the highest hemorrhage risk, while combinations with cilostazol were the lowest. Apixaban, alone and in combination, was associated with reduced hemorrhage and mortality risks. **Conclusions:** Combining anticoagulants with antiplatelets increases hemorrhage and mortality risk. While our findings highlight potential safety signals related to hemorrhage with antithrombotic drug combinations, they remain hypothesis-generating and should not be interpreted as causal associations. Instead, they provide an initial basis for further validation in well-designed clinical cohorts where comorbidities can be adequately accounted for.

## 1. Introduction

The combination of anticoagulants and antiplatelet agents is frequently observed in clinical practice. A cross-sectional study involving 4557 members of Kaiser Permanente Colorado revealed that the prevalence of concurrent warfarin and any antiplatelet therapy was 385 per 1000 patients (95% confidence interval [CI] ranging from 371 to 399 per 1000) [1]. This study also noted that such combinations were more prevalent among males and patients with concomitant heart failure or a history of stroke or transient ischemic attacks [1]. A prospective registry-based cohort study of 1844 patients indicated that those receiving combination therapy with warfarin and aspirin had a prevalence of 26% (95% CI: 23.8–28.3%) compared to 20.3% (95% CI: 18.3–22.3%) among those receiving warfarin alone. Furthermore, the incidence of major bleeding was significantly higher in the combination group at 5.7% (95% CI: 4.6–7.1%) compared to 3.3% (95% CI: 2.4–4.3%) in the warfarin-only group [2]. The European Society of Cardiology (ESC) and the American College of Cardiology/American Heart Association (ACC/AHA) recommend the addition of aspirin to warfarin therapy for patients with atrial fibrillation who have experienced an ischemic event or are undergoing coronary revascularization procedures [3,4]. However, a recent study revealed that inappropriate combinations of warfarin and aspirin were present in 29.5% of patients who experienced major bleeding episodes [5]. Similarly, Turan et al. found that the combination of warfarin and aspirin was prescribed to 38.8% of patients with concomitant vascular disease, 36.6% with diabetes, and 33.3% with left ventricular systolic dysfunction, none of whom had a history of acute coronary syndrome or revascularization procedures [6].

A recent meta-analysis encompassing both randomized clinical trials (RCTs) and observational studies demonstrated an increased risk of bleeding associated with the combined use of oral anticoagulants and aspirin, with an odds ratio (OR) of 1.36 (95% CI: 1.15–1.59) for RCTs and an OR of 1.42 (95% CI: 1.09–1.87) for observational studies. Additionally, this study reported a significantly higher risk of bleeding in RCTs for combination therapy (OR: 2.36; 95% CI: 1.91–2.92), though no significant difference was observed in observational studies (OR: 1.93; 95% CI: 0.99–3.75) [7].

Despite the well-documented risks associated with traditional anticoagulants, there is a lack of data comparing bleeding risks between novel oral anticoagulants (NOACs) such as dabigatran, edoxaban, apixaban and rivaroxaban, and aspirin. An RCT comparing dabigatran with aspirin in 375 patients with cryptogenic stroke found no significant difference in bleeding risk, with an annualized bleeding rate of 3.9% for dabigatran versus 3.5% for aspirin [8]. Conversely, another study involving non-valvular atrial fibrillation patients indicated that dabigatran had the lowest risk of major bleeding episodes, including gastrointestinal and intracranial hemorrhages, compared to warfarin and rivaroxaban when combined with antiplatelet drugs [9]. Another meta-analysis showed that rivaroxaban doses less than 15 mg/day carried a similar bleeding risk to aspirin, while doses greater than 15 mg/day were associated with a higher risk of intracranial hemorrhage [10]. A network meta-analysis further revealed comparable bleeding risks between apixaban and aspirin, whereas other NOACs exhibited higher bleeding risks [11]. Thus, there remains a significant gap in evidence regarding the bleeding risks associated with various anticoagulant-antiplatelet combinations. Furthermore, various antiplatelet agents, including clopidogrel, prasugrel, cilostazol, dipyridamole, and ticagrelor, are utilized in managing thromboembolic disorders, yet literature on the bleeding risks of these combinations with anticoagulants is sparse.

The United States Food and Drug Administration (USFDA) Adverse Event Reporting System (AERS) serves as a publicly available collection of adverse event reports submitted by healthcare personnel through a spontaneous reporting system [12]. USFDA AERS is a valuable tool for identifying signals related to adverse drug events and may guide appropriate monitoring and safety measures [13]. A recent analysis of 33 reports from the World Health Organization’s Vigibase highlighted a higher risk of bleeding in individuals over 75 years of age, with 31 reported fatalities for combined dabigatran and aspirin [14]. Disproportionality analysis, a statistical method comparing the occurrence of adverse events associated with specific drugs to those associated with other drugs, can provide critical insights into potential risks [15]. It is unknown whether any significant differences exist in the risks of hemorrhage between various anticoagulant-antiplatelet drug combinations. Considering this existing gap in the literature, we conducted the present study to comprehensively assess the risks of hemorrhage associated with various combinations of antiplatelet and anticoagulant (including NOACs) drugs using data from the USFDA AERS.

## 2. Methods

### 2.1. Data Source

We extracted data from the USFDA AERS using the Standardized Medical Dictionary for Regulatory Activities Query (SMQ) code 20000038 termed “haemorrhages” [16]. According to MedDRA, this SMQ (broad) has been defined as “Haemorrhage is defined as the escape of blood from the vessels; bleeding. Small haemorrhages are classified according to size as petechiae (very small), purpura (up to 1 cm), and ecchymoses (larger). A large accumulation of blood within a tissue is called a hematoma” [16]. The list of preferred terms (PTs) included in this SMQ (broad) query is provided in Supplementary Table S1. The data collection spanned between March 2004 and June 2024, encompassing 82 quarters.

### 2.2. Data Processing

The USFDA AERS database was queried using the above SMQ without any restrictions to demographic characteristics for fetching individual case safety reports [17]. The anticoagulant drugs included in the search query were as follows: Acenocoumarol, apixaban, betrixaban, dabigatran, edoxaban, rivaroxaban and warfarin. The following antiplatelet drugs were queried: aspirin, cilostazol, clopidogrel, dipyridamole, prasugrel and ticagrelor. Reporting pertaining to hemorrhage was obtained individually for the above drugs as well as for each anticoagulant-antiplatelet pair. We adhered to the USFDA recommendations for identifying and eliminating duplicate reports. We sorted out the reports using the Case_ID, following which the reports were arranged in chronological order, with the report dated latest that also contained the highest FDA_DT (or Individual Safety Report number) was retained, excluding the older reports. The USFDA assigns the role of the drug with the specific adverse event into one of the following categories: primary suspect, secondary suspect, interacting, or concomitant. We retained only the reports with a primary suspect role (for queries related to individual antiplatelet and anticoagulant drugs) and names of the drugs specified in non-proprietary format. The deduplication process was performed separately for each drug/drug pair. Demographic characteristics, including age, gender, year, and country of reporting, were obtained for drugs that showed positive signals.

### 2.3. Data Mining Algorithms

We used the “case-non-case” approach in the disproportionality analysis for signal detection between the anticoagulant, antiplatelet and anticoagulant-antiplatelet drugs/drug pairs with hemorrhage. This approach involves comparing the occurrence of hemorrhage with those with exposure to anticoagulants or antiplatelets or combined anticoagulants with antiplatelets in comparison to non-cases (other reported events for the drug/s of interest) [18]. We used the Openvigil 2.1 platform for obtaining the relevant data on drug(s)–event pairs, including demographic details. We used frequentist and Bayesian approaches for signal detection that included four data mining algorithms. The Reporting Odds Ratio (ROR) and the Proportional Reporting Ratio (PRR) constituted the frequentist methods. The PRR is calculated as the ratio of the proportion of hemorrhage reports among all adverse events for a given drug to the proportion of hemorrhage reports for all other drugs in the database; a PRR ≥ 2 suggests that the event is reported at least twice as often as expected. The ROR is derived from a two-by-two contingency table of cases versus non-cases for the drug of interest, expressed as the odds of reporting hemorrhage with the drug compared to the odds with all other drugs. In pharmacovigilance, PRR and ROR serve as relative measures of disproportionality that indicate whether a specific drug-event pair is reported more frequently than expected by chance, analogous in interpretation to relative risk or odds ratio in epidemiological studies. Evan’s criteria were employed for signal detection in the frequentist approach, where the following criteria had to be met: the presence of at least three reports, PRR ≥ 2, and Chi-square (χ^2^) value ≥ 4 for each drug(s)–hemorrhage pair [19]. The ROR was calculated with a 95% CI, and it was considered significant when the lower limit of the CI exceeded one. The pharmacovigilance equivalent of the relative risk used in other study designs for indicating the relative occurrence of events is the PRR. These measures do not provide incidence rates but rather highlight disproportional reporting, which serves as an initial signal warranting further evaluation.

Amongst the Bayesian approaches, we included the Bayesian Confidence Propagation Neural Network (BCPNN) and the Multi-Item Gamma Poisson Shrinker (MGPS). In BCPNN, the signal detection measure used was the Information Component (IC) that represents the logarithmic ratio of the joint probability of anticoagulant/antiplatelet drug or combined anticoagulant-antiplatelet drugs with hemorrhage compared to the product of their individual probabilities obtained with the data reported in the USFDA AERS database. When the lower limit of the 95% CI of IC (IC025) exceeded zero, a signal was generated. In the MGPS method, the Empirical Bayes Geometric Mean (EBGM) was calculated, and when the lower limit of the 95% CI (EBGM05) exceeded 2, a signal was generated.

The outcomes in the unique reports were categorized into one of the following: death, disability, or hospitalization (initial or prolonged). We adhered to the REporting of A Disproportionality analysis for DrUg Safety signal detection using spontaneously reported adverse events in Pharmacovigilance (READUS-PV) guidelines [20].

### 2.4. Statistical Analysis

Descriptive statistics were used to represent demographic variables. Means (SD) were used for calculating numerical variables, while proportions (%) were used for categorical variables. The Chi-square test (χ^2^) was employed to assess the significance of outcome distributions across drug/drug combinations for hemorrhage. The statistical analyses were performed using SPSS (IBM Corp. Released 2020. IBM SPSS Statistics for Windows, Version 27.0. Armonk, NY, USA: IBM Corp.). ChatGPT 4.0 was used for enhancing the language and improving the grammar.

## 3. Results

### 3.1. Search Results

**Figure 1:**
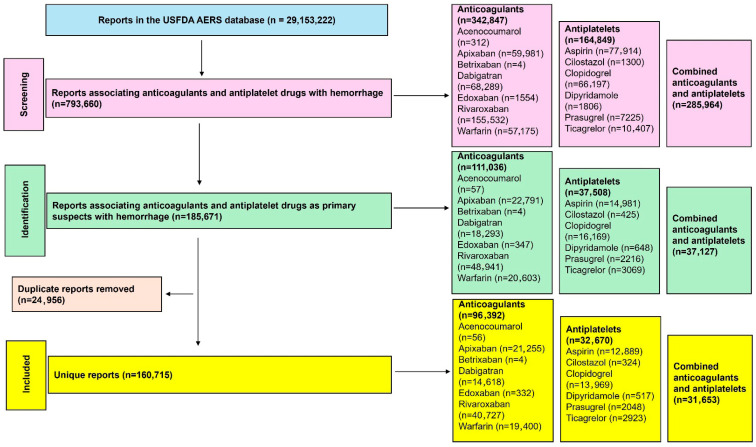
Study flow diagram. A total of 160,715 unique reports were included in this study for the analysis of the risk of hemorrhage.

A total of 29,153,222 reports were present in the USFDA AERS database, of which 160,715 were included in this study (Figure 1). Anticoagulants had a greater number of reports than antiplatelet drugs, with rivaroxaban, followed by apixaban, warfarin, and dabigatran, contributing the most reports among the anticoagulants. Among the antiplatelet drugs, clopidogrel, followed by aspirin, was the most frequently reported. The detailed number of unique reports with various combinations of anticoagulants and antiplatelet drugs is provided in Supplementary Table S2. In reports related to combination therapies, anticoagulants were most often combined with aspirin, followed by clopidogrel. Only four reports of hemorrhage were associated with betrixaban, and there were no reports of hemorrhage with the combined use of betrixaban and any antiplatelet drug. No further analysis was performed for betrixaban. Similarly, no reports associated hemorrhage with combined edoxaban and dipyridamole or cilostazol, and only one report was associated with acenocoumarol and prasugrel.

### 3.2. Signal Detection Measures with Anticoagulants and Antiplatelets Alone

**Figure 2:**
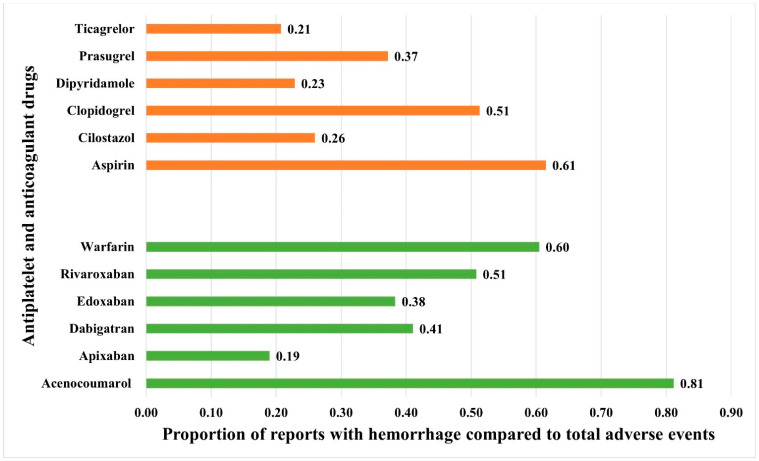
Proportion of spontaneously filed reports with hemorrhage associated with anticoagulants and antiplatelets. The horizontal bars represent the proportions of reports with hemorrhage compared to total reported adverse events with antiplatelets (red bars) and anticoagulants (green bars).

**Figure 3:**
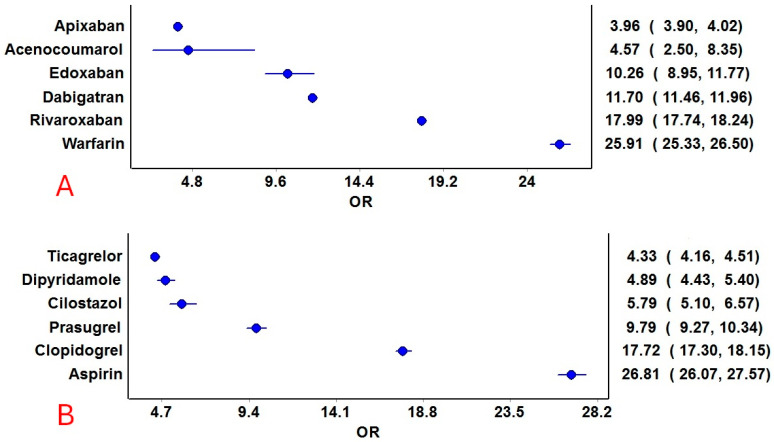
Reporting odds ratios of anticoagulant and antiplatelet drugs. (**A**): Anticoagulants and (**B**): Antiplatelet drugs. The blue circles represent the reporting odds ratio (ROR), and the horizontal blue lines represent the 95% CI for ROR.

Hemorrhage was reported with all anticoagulants and antiplatelet drugs. Among the anticoagulants, acenocoumarol, followed by warfarin, had the highest prevalence of hemorrhage among all the reported adverse events. Among the antiplatelets, aspirin followed by clopidogrel had the highest prevalence in relation to the total number of adverse event reports (Figure 2). The ROR [95% CI] for hemorrhage associated with anticoagulants and antiplatelets is presented in Figure 3. Despite many reports (n = 21,255), apixaban showed a relatively lower ROR and PRR compared to other anticoagulants. Warfarin, followed by rivaroxaban, had the highest RORs among the anticoagulants, while aspirin, followed by clopidogrel, had the highest RORs among the antiplatelets. Other frequentist and Bayesian measures are summarized in Table 1. The demographic characteristics from reports involving anticoagulants and hemorrhage are summarized in Supplementary Tables S3–S8, and for antiplatelet drugs in Supplementary Tables S9–S14. Most patients were in the elderly category with no gender predilection.

### 3.3. Signal Detection Measures with Combined Anticoagulant and Antiplatelet Drugs

**Figure 4:**
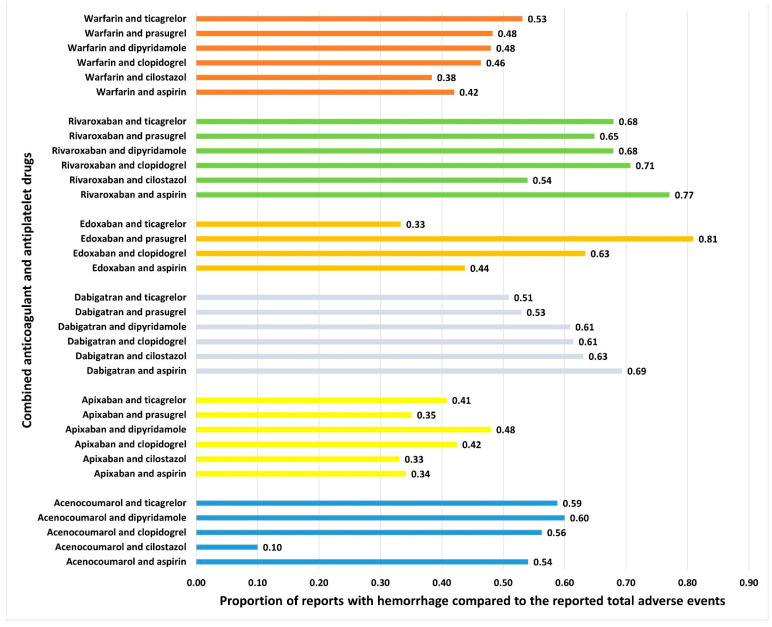
Proportion of spontaneously filed reports with hemorrhage associated with combined anticoagulants and antiplatelets. The horizontal bars represent the proportions of reports with hemorrhage compared to total reported adverse events with combined antiplatelets and anticoagulants.

**Figure 5:**
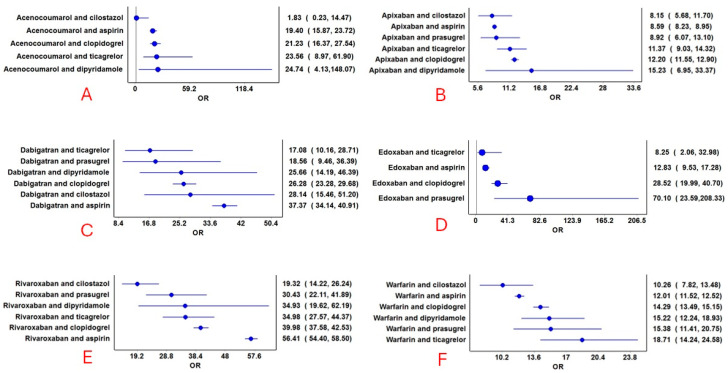
Reporting odds ratios of combined anticoagulant and antiplatelet drugs. (**A**): Acenocoumarol combinations with antiplatelets; (**B**): Apixaban combinations with antiplatelets; (**C**): Dabigatran combinations with antiplatelets; (**D**): Edoxaban combinations with antiplatelets; (**E**): Rivaroxaban combinations with antiplatelets; and (**F**): Warfarin combinations with antiplatelets. The blue circles represent the reporting odds ratio (ROR), and the horizontal blue lines represent the 95% CI for ROR.

Hemorrhage was reported with all combinations of anticoagulant and antiplatelet drugs (Figure 4). No specific pattern emerged, except those combinations of anticoagulants (except dabigatran) with cilostazol and apixaban combinations with antiplatelet drugs had a lower proportion of hemorrhage reports compared to other drug combinations. The RORs for various combinations are presented in Figure 5. Although the combination of edoxaban and prasugrel had the highest ROR, only 17 cases were reported. Rivaroxaban combined with aspirin (n = 12,801), followed by dabigatran combined with aspirin (n = 1533), had relatively higher RORs. Combinations of cilostazol with anticoagulants (except dabigatran) exhibited the lowest RORs. Additional frequentist and Bayesian measures are summarized in Table 2. Apixaban combinations revealed a potentially lower ROR and PRR for the risk of hemorrhage. The frequentist measures were positive for all combinations except acenocoumarol with cilostazol, while Bayesian measures were positive for all combinations except acenocoumarol with cilostazol and dipyridamole, and edoxaban with ticagrelor. Demographic characteristics for various anticoagulant and antiplatelet drug combinations are summarized in Supplementary Tables S15–S48. In general, most patients were in the elderly age category and were statistically significant compared to other age groups (χ^2^: 765.6; *p* < 0.00001). Statistically significant differences were observed in the distribution of bleeding based on gender (anticoagulants: males, 53%; antiplatelets: males, 59%; and combined anticoagulants and antiplatelets: males, 61%; χ^2^: 603.7; *p* < 0.0001).

### 3.4. Comparison of Reported Outcome Measures

**Figure 6:**
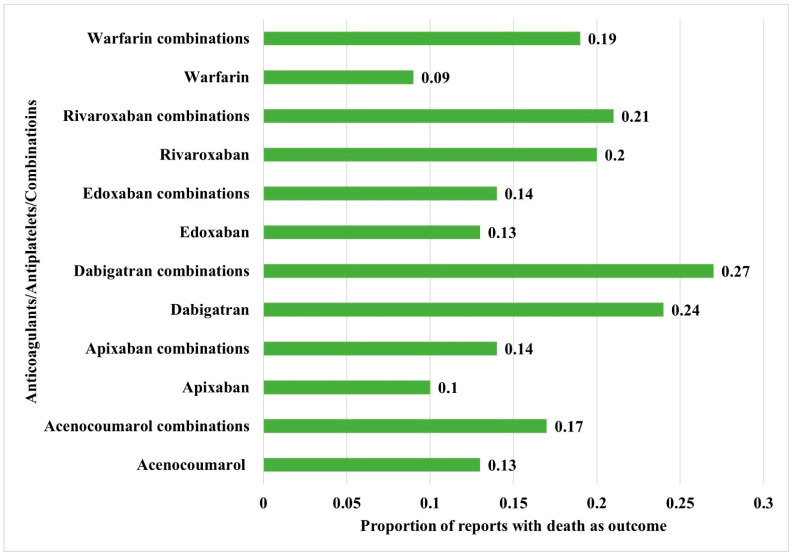
Comparison of mortality between the anticoagulants alone and their combinations with antiplatelet drugs. The horizontal bars represent the proportion of reports with death as an outcome compared to the total reports.

Table 3 shows the distribution of key outcomes between anticoagulants, antiplatelets, and their combinations. For all drugs and drug combinations, most hemorrhages resulted in hospitalization (initial or prolonged), followed by death and life-threatening outcomes. Statistically significant differences in outcomes were reported between anticoagulants, antiplatelets, and combinations involving dabigatran, rivaroxaban, and warfarin with antiplatelet drugs. Distributions of the proportion of reports with death as outcomes between the anticoagulants and their combinations with antiplatelet drugs are depicted in Figure 6 and were significantly different between the interventions (χ^2^: 2622.9; df: 11; *p* < 0.05). The use of combined anticoagulants and antiplatelets was associated with a higher risk of mortality compared to anticoagulants alone. Apixaban, both on its own and in combination with antiplatelet drugs, showed a potentially lower risk of hemorrhage, along with a reduced risk of mortality.

## 4. Discussion

### 4.1. Key Outcomes

The analysis of 160,715 reports from the USFDA AERS database revealed that anticoagulants, particularly rivaroxaban, apixaban, warfarin, and dabigatran, had more reports than antiplatelet drugs, with clopidogrel and aspirin contributing the most among the latter. Hemorrhage was commonly reported with both anticoagulants and antiplatelets, with acenocoumarol and warfarin showing the highest prevalence among anticoagulants, and aspirin and clopidogrel among antiplatelets. Apixaban had the least ROR amongst anticoagulants for hemorrhage. Also, apixaban combinations revealed potentially lower RORs for hemorrhage. Combinations of anticoagulants with antiplatelet drugs, especially rivaroxaban and dabigatran with aspirin, were associated with higher RORs for hemorrhage, and apixaban combinations revealed relatively lower RORs. Notably, combinations involving cilostazol had fewer hemorrhage reports and lower RORs. Hospitalization, death, and life-threatening outcomes were the most frequent consequences across all drug categories. The use of combined anticoagulants and antiplatelets was associated with a higher risk of mortality compared to anticoagulants alone. Apixaban, both on its own and in combination with antiplatelet drugs, showed a potentially lower risk of hemorrhage, along with a reduced risk of mortality.

### 4.2. Comparison with Existing Literature

All anticoagulant–antiplatelet combinations were associated with disproportional signals, suggesting higher reporting rates of hemorrhage and mortality compared to monotherapies. Among anticoagulants, apixaban consistently showed a lower reporting signal (based on RORs) for both hemorrhage and mortality, whether used alone or with antiplatelets. In the ARISTOTLE trial, apixaban was associated with lower rates of major bleeding (2.13% vs. 3.09% per year; HR 0.69, 95% CI 0.60–0.80) and all-cause mortality (3.52% vs. 3.94%; HR 0.89, 95% CI 0.80–0.99) compared to warfarin [16]. A meta-analysis of 267,998 atrial fibrillation patients similarly found a lower bleeding risk with apixaban (RR 0.63; 95% CI 0.58–0.68), though no difference in all-cause mortality [17].

By contrast, rivaroxaban showed the highest disproportionality signal for hemorrhage. Real-world comparisons found rivaroxaban carried a higher bleeding risk (HR 1.35; 95% CI 1.26–1.45) and hospitalization for bleeding (HR 1.43; 95% CI 1.17–1.74) compared to apixaban [18]. Annual bleeding incidence was 11% with apixaban, 13.5% with dabigatran, and 53.5% with rivaroxaban [18]. Another study found rivaroxaban had a similar bleeding risk to warfarin (HR 0.98; 95% CI 0.83–1.17), while apixaban and dabigatran were safer [20]. Apixaban also demonstrated lower gastrointestinal bleeding risk compared to dabigatran (HR 0.39; 95% CI 0.27–0.58) and rivaroxaban (HR 0.33; 95% CI 0.22–0.49), with consistency across elderly subgroups [21]. In a large cohort of older adults, major ischemic or hemorrhagic events were lower with apixaban than rivaroxaban (13.4 vs. 16.1 per 1000 person-years) [22]. A 5-year claims analysis also showed higher bleeding rates with warfarin (6 per 100 person-years) and rivaroxaban (5 per 100 person-years) compared to apixaban (3.3 per 100 person-years) and dabigatran (2.8 per 100 person-years) [23].

Our study also confirmed that anticoagulant–antiplatelet combinations carried stronger bleeding and mortality signals than monotherapy. Prior data similarly showed increased bleeding and mortality risk with combined therapy versus dual antiplatelet regimens post-stenting [24]. In atrial fibrillation, apixaban demonstrated lower or comparable bleeding risks compared with aspirin [25] or warfarin [26] and was associated with significant absolute risk reduction in patients receiving antiplatelets after ACS or PCI [27].

Taken together, these findings highlight potential differences in anticoagulant safety, with apixaban generally demonstrating a favorable profile in both our signal analysis and clinical studies [16,28,29]. Clinical risk assessment using validated tools (ATRIA, HAS-BLED, DOAC scores) remains essential [30,31], particularly as bleeding risk is strongly influenced by patient-specific comorbidities. It is important to note that while our analysis found strong disproportionality signals for rivaroxaban and warfarin, large RCTs such as ROCKET-AF reported no significant difference in clinically relevant bleeding between rivaroxaban and warfarin [32], underscoring the distinction between spontaneous reporting data and incidence-based trial results. Likewise, while warfarin showed higher signals than edoxaban in our study, the ENTRUST-AF PCI trial found edoxaban non-inferior to warfarin for bleeding [33]. These discrepancies reinforce the need to interpret pharmacovigilance signals cautiously and in the context of trial evidence.

Bleeding risk with antithrombotic therapy is especially concerning in multimorbid, frail, and elderly patients. Such groups face higher vulnerability due to impaired drug clearance, polypharmacy, and reduced physiological reserve. Even minor bleeding can lead to hospitalization, disability, or death. While the AERS database lacks granularity to stratify risk by frailty or multimorbidity, the strong signals observed emphasize the need for prospective studies in these high-risk populations. Age-related changes and comorbidities likely explain why most bleeding reports involved elderly patients. The higher bleeding rates among males observed here are also notable, consistent with sex-related biological and prescribing differences [34].

### 4.3. Strengths and Limitations

This study has several strengths, including the use of a large dataset from the USFDA AERS database, with 160,715 relevant reports from over 29 million cases, offering a comprehensive view of anticoagulant and antiplatelet-associated hemorrhages. The inclusion of a wide range of drugs allows for detailed comparisons, while the application of both frequentist and Bayesian methods enhances the robustness of signal detection. Additionally, the focus on combination therapies provides novel insights into drug interactions, and the detailed analysis of clinical outcomes such as hospitalization and mortality adds clinical relevance to the findings. This study has several important limitations. First, as with any pharmacovigilance analysis based on a spontaneous reporting system, it is subject to inherent biases, including underreporting, selective reporting, and variable data quality. The absence of denominator data prevents the calculation of absolute incidence rates. Second, many reports in the USFDA AERS database contain incomplete or missing clinical information, limiting the ability to characterize patients fully. A critical limitation is the inability to account for important confounding factors such as comorbidities (e.g., chronic kidney disease, liver dysfunction, hypertension, or prior bleeding history) and concomitant medications, all of which can significantly alter hemorrhage risk. For example, advanced chronic kidney disease is associated with uremia-induced platelet dysfunction, impaired coagulation, and altered drug clearance, markedly amplifying bleeding risk in patients receiving antithrombotic therapy. Similarly, hepatic dysfunction reduces the synthesis of clotting factors, further predisposing patients to hemorrhage. Third, data on newer antithrombotic agents such as betrixaban and edoxaban remain relatively sparse, limiting the robustness of signal detection for these drugs. Our study is also limited by its characterization of hemorrhage as a composite outcome without sub-classification into distinct clinical categories (such as gastrointestinal or intracranial). Consequently, the results represent a composite risk across hemorrhage types of varying severity, which may constrain specific clinical interpretations, limiting the specificity of the study findings. Taken together, these limitations underscore that our analysis should be interpreted as a hypothesis- and signal-generating effort. Future research using prospective cohorts, electronic health record–linked datasets, and clinical trials is warranted to validate these findings, explore dose–response relationships, and consider the influence of pharmacogenomic and patient-specific factors to better inform individualized therapy. Importantly, the overarching clinical challenge lies in balancing the efficacy of antithrombotic therapy in preventing ischemic and thromboembolic events against the heightened risk of bleeding. Our results should, therefore, be viewed as complementary to this broader therapeutic context, supporting the need for individualized management strategies rather than a one-size-fits-all approach. Moving forward, prospective studies or clinical trials are needed to validate these findings, and future research should explore the risk-benefit balance of combination therapies, especially in high-risk patient populations. The incorporation of real-world data, such as electronic health records, would enable more accurate incidence estimates and adjustment for confounders. Moreover, studies focusing on dose–response relationships and pharmacogenomics could offer deeper insights into hemorrhage risks and support personalized treatment approaches.

## 5. Conclusions

This study provides a comprehensive analysis of anticoagulant and antiplatelet-associated hemorrhages using data from the USFDA AERS database. Anticoagulants, particularly warfarin and rivaroxaban, as well as antiplatelet agents like aspirin and clopidogrel, were commonly associated with hemorrhagic events. The findings suggest important disproportionality signals that warrant close monitoring of patients on these therapies, especially when used in combination. Apixaban, both on its own and in combination with antiplatelet drugs, showed a potentially lower risk of hemorrhage, along with a reduced risk of mortality. However, these signals do not equate to incidence rates, and clinical recommendations should, therefore, remain cautious. While the study highlights significant safety signals, the limitations of spontaneous reporting data and the absence of true incidence rates call for cautious interpretation of the results. Importantly, this analysis is signal-generating only and does not allow for causal inference. While our findings highlight potential safety signals related to hemorrhage with antithrombotic drug combinations, they should be viewed strictly as preliminary associations that require confirmation in prospective studies where comorbidities such as CKD, liver disease, and prior bleeding history can be adequately accounted for. Future research should focus on prospective studies and real-world data to further assess the safety of these therapies and guide clinical decision-making. Ultimately, this analysis emphasizes the need for individualized patient management to balance the therapeutic benefits of anticoagulant and antiplatelet drugs against their potential risks.

## Figures and Tables

**Table 1:** Signal detection measures for anticoagulants and antiplatelets.

Anticoagulants and Antiplatelets	RRR	PRR	95% Lower Limit PRR	95% Upper Limit PRR	Signal by Frequentist Approach	Number of Reports	IC025	EBGM05	Signal by Bayesian Approach
Acenocoumarol	14.2	14.2	12.7	15.9	Positive	56	2.1	7.8	Positive
Apixaban	3.3	3.4	3.3	3.5		21,255	1.7	3.3	
Dabigatran	7.2	7.3	7.2	7.4		14,618	2.8	7	
Edoxaban	6.7	6.7	6.2	7.3		332	2.4	5.8	
Rivaroxaban	8.9	9.4	9.3	9.4		40,727	3.1	8.8	
Warfarin	10.6	10.8	10.7	10.9		19,400	3.3	10.3	
Antiplatelets									
Aspirin	10.8	10.9	10.8	11.1	Positive	12,889	3.3	10.5	Positive
Cilostazol	4.5	4.5	5.1	5		324	1.9	4	
Clopidogrel	9	9.1	9	9.2		13,970	3.1	8.8	
Dipyridamole	4	4	3.7	4.3		517	1.8	3.6	
Prasugrel	6.5	6.5	5.3	6.8		2048	2.6	6.2	
Ticagrelor	3.6	3.6	3.5	3.8		2922	1.8	3.5	

PRR: Proportional reporting ratio; RRR: Relative reporting ratio; IC: Information component; EBGM: Empirical Bayes geometric mean.

**Table 2:** Signal detection measures for combined anticoagulants and antiplatelets.

Anticoagulants and Antiplatelets	RRR	PRR	95% Lower Limit PRR	95% Upper Limit PRR	Signal by Frequentist Approach	Number of Reports	IC025	EBGM05	Signal by Bayesian Approach
Acenocoumarol combinations									
Aspirin	9.5	9.5	8.6	10.4	207	Positive	2.7	7.7	Positive
Cilostazol	1.7	1.7	0.3	11.2	1	Negative	0.1	0.2	Negative
Clopidogrel	9.8	9.8	8.8	11	130	Positive	2.5	7.6	Positive
Dipyridamole	10.5	10.5	5.1	21.5	3		0.6	1.8	Negative
Ticagrelor	10.3	10.3	6.9	15.3	10		1.3	3.9	Positive
Apixaban combinations									
Aspirin	6	6	5.8	6.2	3334	Positive	2.5	5.7	Positive
Cilostazol	5.8	5.8	4.5	7.4	44		1.8	4	
Clopidogrel	7.4	7.4	7.2	7.7	2168		2.7	7	
Dipyridamole	8.4	8.4	5.6	12.6	12		1.4	3.8	
Prasugrel	6.1	6.1	4.8	7.9	40		1.8	4.2	
Ticagrelor	7.1	7.1	6.2	8.2	122		2.3	5.7	
Dabigatran combinations									
Aspirin	12.1	12.2	11.8	12.5	1533	Positive	3.3	11.1	Positive
Cilostazol	11.1	11.1	8.8	13.8	29		1.9	6.1	
Clopidogrel	10.7	10.8	10.3	11.3	675		3.1	9.5	
Dipyridamole	10.6	10.6	8.4	13.4	28		1.9	5.9	
Prasugrel	9.3	9.3	6.7	12.7	18		1.6	4.7	
Ticagrelor	8.9	8.9	6.9	11.5	29		1.9	5.3	
Edoxaban combinations									
Aspirin	7.7	7.7	6.5	9.1	77	Positive	2.2	5.7	Positive
Clopidogrel	11.1	11.1	9.7	12.6	83		2.4	7.8	
Prasugrel	14.2	14.2	11.5	17.4	17		1.3	4.8	
Ticagrelor	5.8	5.8	2.3	14.7	3		0.6	1.5	Negative
Rivaroxaban combinations									
Aspirin	13.5	13.7	13.6	13.8	12,801	Positive	3.6	13	Positive
Cilostazol	9.4	9.4	8.2	10.9	89		2.4	6.9	
Clopidogrel	12.4	12.4	12.2	12.7	3431		3.4	11.6	
Dipyridamole	11.9	11.9	9.9	14.3	36		2	6.7	
Prasugrel	11.4	11.3	10.1	12.7	107		2.5	8.2	
Ticagrelor	11.9	11.9	11.1	12.8	212		2.8	9.4	
Warfarin combinations									
Aspirin	7.4	7.4	7.2	7.6	3842	Positive	2.8	7.1	Positive
Cilostazol	6.7	6.7	5.7	7.9	84		2.1	5.1	
Clopidogrel	8.1	8.1	7.9	8.4	2140		2.9	7.7	
Dipyridamole	8.4	8.4	7.5	9.4	155		2.5	6.7	
Prasugrel	8.4	8.4	7.2	9.9	83		2.3	6.3	
Ticagrelor	9.3	9.3	8.2	10.6	110		2.5	7.1	

PRR: Proportional reporting ratio; RRR: Relative reporting ratio; IC: Information component; EBGM: Empirical Bayes geometric mean.

**Table 3:** Comparison of key reported outcomes between antiplatelets and anticoagulants.

Drug/Drug Combinations	Death	Life-Threatening	Hospitalization	χ^2^ Test Value; df; *p*-Values
Anticoagulants				
Acenocoumarol	7	15	42	2520; 10; <0.05 *
Apixaban	2176	1925	8103	
Dabigatran	3499	1234	10,382	
Edoxaban	42	31	229	
Rivaroxaban	8049	1611	22,403	
Warfarin	1790	1980	11,210	
Antiplatelets				
Aspirin	2129	660	9152	574; 10; <0.05 *
Cilostazol	66	28	132	
Clopidogrel	1704	1158	7089	
Dipyridamole	91	58	236	
Prasugrel	237	190	843	
Ticagrelor	440	359	1233	
Acenocoumarol combinations				
Aspirin	20	46	169	3.8; 8; 0.8
Clopidogrel	0	0	1	
Dipyridamole	11	26	105	
Prasugrel	1	0	2	
Ticagrelor	0	1	3	
Apixaban combinations				
Aspirin	408	339	1766	12.2; 10; 0.3
Cilostazol	8	7	31	
Clopidogrel	292	251	1199	
Dipyridamole	1	0	7	
Prasugrel	5	1	17	
Ticagrelor	21	18	50	
Dabigatran combinations				
Aspirin	294	96	1326	22.4; 10; 0.01 *
Cilostazol	4	1	26	
Clopidogrel	147	55	549	
Dipyridamole	15	0	25	
Prasugrel	6	1	14	
Ticagrelor	6	0	15	
Edoxaban combinations				
Aspirin	6	7	58	9.5; 6; 0.1
Clopidogrel	19	3	61	
Prasugrel	2	0	10	
Ticagrelor	0	0	3	
Rivaroxaban combinations				
Aspirin	2807	520	11,285	67.2; 10; 0.0001 *
Cilostazol	21	6	70	
Clopidogrel	798	253	3265	
Dipyridamole	11	0	33	
Prasugrel	12	6	85	
Ticagrelor	32	19	156	
Warfarin combinations				
Aspirin	579	349	2473	59.6; 10; 0.0001 *
Cilostazol	21	5	55	
Clopidogrel	315	182	1305	
Dipyridamole	30	25	78	
Prasugrel	25	7	44	
Ticagrelor	11	27	66	

*—Statistically significant by Chi-square test.

## Data Availability

The dataset generated during and/or analyzed during the current study are available from the corresponding author on reasonable request.

## Supplementary Materials

The following supporting information can be downloaded at: https://www.mdpi.com/article/10.3390/jcm14176262/s1, Table S1: List of Preferred Terms included in the SMQ (Broad) for hemorrhage; Table S2: Number of reports pertaining to combined anticoagulant and antiplatelet drugs; Table S3: Demographic characteristics in the reports related to hemorrhage with acenocoumarol; Table S4: Demographic characteristics in the reports related to hemorrhage with apixaban; Table S5: Demographic characteristics in the reports related to hemorrhage with dabigatran; Table S6: Demographic characteristics in the reports related to hemorrhage with edoxaban; Table S7: Demographic characteristics in the reports related to hemorrhage with rivaroxaban; Table S8: Demographic characteristics in the reports related to hemorrhage with warfarin; Table S9: Demographic characteristics in the reports related to hemorrhage with aspirin; Table S10: Demographic characteristics in the reports related to hemorrhage with cilostazol; Table S11: Demographic characteristics in the reports related to hemorrhage with clopidogrel; Table S12: Demographic characteristics in the reports related to hemorrhage with dipyridamole; Table S13: Demographic characteristics in the reports related to hemorrhage with prasugrel; Table S14: Demographic characteristics in the reports related to hemorrhage with ticagrelor; Table S15: Demographic characteristics in the reports related to hemorrhage with acenocoumarol with aspirin; Table S16: Demographic characteristics in the reports related to hemorrhage with acenocoumarol with cilostazol; Table S17: Demographic characteristics in the reports related to hemorrhage with acenocoumarol with clopidogrel; Table S18: Demographic characteristics in the reports related to hemorrhage with acenocoumarol with dipyridamole; Table S19: Demographic characteristics in the reports related to hemorrhage with acenocoumarol with prasugrel; Table S20: Demographic characteristics in the reports related to hemorrhage with acenocoumarol with ticagrelor; Table S21: Demographic characteristics in the reports related to hemorrhage with apixaban with aspirin; Table S22: Demographic characteristics in the reports related to hemorrhage with apixaban with cilostazol; Table S23: Demographic characteristics in the reports related to hemorrhage with apixaban with clopidogrel; Table S24: Demographic characteristics in the reports related to hemorrhage with apixaban with dipyridamole; Table S25: Demographic characteristics in the reports related to hemorrhage with apixaban with prasugrel; Table S26: Demographic characteristics in the reports related to hemorrhage with apixaban with ticagrelor; Table S27: Demographic characteristics in the reports related to hemorrhage with dabigatran with aspirin; Table S28: Demographic characteristics in the reports related to hemorrhage with dabigatran with cilostazol; Table S29: Demographic characteristics in the reports related to hemorrhage with dabigatran with clopidogrel; Table S30: Demographic characteristics in the reports related to hemorrhage with dabigatran with dipyridamole; Table S31: Demographic characteristics in the reports related to hemorrhage with dabigatran with prasugrel; Table S32: Demographic characteristics in the reports related to hemorrhage with dabigatran with ticagrelor; Table S33: Demographic characteristics in the reports related to hemorrhage with edoxaban with aspirin; Table S34: Demographic characteristics in the reports related to hemorrhage with edoxaban with clopidogrel; Table S35: Demographic characteristics in the reports related to hemorrhage with edoxaban with prasugrel; Table S36: Demographic characteristics in the reports related to hemorrhage with edoxaban with ticagrelor; Table S37: Demographic characteristics in the reports related to hemorrhage with rivaroxaban with aspirin; Table S38: Demographic characteristics in the reports related to hemorrhage with rivaroxaban with cilostazol; Table S39: Demographic characteristics in the reports related to hemorrhage with rivaroxaban with clopidogrel; Table S40: Demographic characteristics in the reports related to hemorrhage with rivaroxaban with dipyridamole; Table S41: Demographic characteristics in the reports related to hemorrhage with rivaroxaban with prasugrel; Table S42: Demographic characteristics in the reports related to hemorrhage with rivaroxaban with ticagrelor; Table S43: Demographic characteristics in the reports related to hemorrhage with warfarin with aspirin; Table S44: Demographic characteristics in the reports related to hemorrhage with warfarin with cilostazol; Table S45: Demographic characteristics in the reports related to hemorrhage with warfarin with clopidogrel; Table S46: Demographic characteristics in the reports related to hemorrhage with warfarin with dipyridamole; Table S47: Demographic characteristics in the reports related to hemorrhage with warfarin with prasugrel; Table S48: Demographic characteristics in the reports related to hemorrhage with warfarin with ticagrelor.
