# Supplementary material for: Hemorrhage Risk Associated with Anticoagulant and Antiplatelet Drug Combinations: Insights from the USFDA Adverse Event Reporting System
Source: J Clin Med. 2025 Sep 4;14(17):6262. doi: 10.3390/jcm14176262 (PMC12429624; doi:10.3390/jcm14176262)
Supplement: Supplementary file 1 [file jcm-14-06262-s001.zip › jcm-3837433-supplementary.pdf]

Supplementary Table S1. List of Preferred Terms included in the SMQ (Broad) for hemorrhage.

MedDRA code	Preferred Term
10085424	Abnormal uterine bleeding
10069195	Abnormal withdrawal bleeding
10069195	Abnormal withdrawal bleeding
10067817	Acute haemorrhagic conjunctivitis
10067817	Acute haemorrhagic conjunctivitis
10067817	Acute haemorrhagic conjunctivitis
10058994	Acute haemorrhagic leukoencephalitis
10058994	Acute haemorrhagic leukoencephalitis
10070599	Acute haemorrhagic oedema of infancy
10070599	Acute haemorrhagic oedema of infancy
10070599	Acute haemorrhagic oedema of infancy
10070599	Acute haemorrhagic oedema of infancy
10075634	Acute haemorrhagic ulcerative colitis
10075634	Acute haemorrhagic ulcerative colitis
10057373	Adenoviral haemorrhagic cystitis
10057373	Adenoviral haemorrhagic cystitis
10064223	Angina bullosa haemorrhagica
10064223	Angina bullosa haemorrhagica
10005104	Bleeding anovulatory
10005104	Bleeding anovulatory

10049227	Bleeding time abnormal
10005140	Bleeding time prolonged
10005144	Bleeding varicose vein
10083809	Bullous haemorrhagic dermatosis
10083809	Bullous haemorrhagic dermatosis
10008086	Cerebral arteriovenous malformation haemorrhagic
10008086	Cerebral arteriovenous malformation haemorrhagic
10008086	Cerebral arteriovenous malformation haemorrhagic
10050399	Chronic gastrointestinal bleeding
10050399	Chronic gastrointestinal bleeding
10065019	Coital bleeding
10051558	Corneal bleeding
10051558	Corneal bleeding
10011793	Cystitis haemorrhagic
10012741	Diarrhoea haemorrhagic
10012741	Diarrhoea haemorrhagic
10013541	Diverticulitis intestinal haemorrhagic
10013541	Diverticulitis intestinal haemorrhagic
10013541	Diverticulitis intestinal haemorrhagic
10013560	Diverticulum intestinal haemorrhagic
10013560	Diverticulum intestinal haemorrhagic
10013865	Duodenitis haemorrhagic
10013865	Duodenitis haemorrhagic

10014589	Encephalitis haemorrhagic
10014896	Enterocolitis haemorrhagic
10014896	Enterocolitis haemorrhagic
10053196	Eyelid bleeding
10053196	Eyelid bleeding
10053196	Eyelid bleeding
10017857	Gastritis alcoholic haemorrhagic
10017857	Gastritis alcoholic haemorrhagic
10017866	Gastritis haemorrhagic
10017866	Gastritis haemorrhagic
10080561	Gastrointestinal vascular malformation haemorrhagic
10080561	Gastrointestinal vascular malformation haemorrhagic
10018276	Gingival bleeding
10018276	Gingival bleeding
10079902	Haemorrhagic adrenal infarction
10079902	Haemorrhagic adrenal infarction
10064595	Haemorrhagic arteriovenous malformation
10064595	Haemorrhagic arteriovenous malformation
10059766	Haemorrhagic ascites
10059766	Haemorrhagic ascites
10059766	Haemorrhagic ascites
10077443	Haemorrhagic breast cyst
10077443	Haemorrhagic breast cyst

10077443	Haemorrhagic breast cyst
10085944	Haemorrhagic cerebellar infarction
10085944	Haemorrhagic cerebellar infarction
10019005	Haemorrhagic cerebral infarction
10019005	Haemorrhagic cerebral infarction
10082088	Haemorrhagic cholecystitis
10082088	Haemorrhagic cholecystitis
10059189	Haemorrhagic cyst
10059189	Haemorrhagic cyst
10059189	Haemorrhagic cyst
10062713	Haemorrhagic diathesis
10062713	Haemorrhagic diathesis
10019008	Haemorrhagic disease of newborn
10019008	Haemorrhagic disease of newborn
10019008	Haemorrhagic disease of newborn
10019008	Haemorrhagic disease of newborn
10019009	Haemorrhagic disorder
10019009	Haemorrhagic disorder
10067786	Haemorrhagic erosive gastritis
10067786	Haemorrhagic erosive gastritis
10075015	Haemorrhagic fever with renal syndrome
10085136	Haemorrhagic gastroenteritis
10085136	Haemorrhagic gastroenteritis

10067796	Haemorrhagic hepatic cyst
10067796	Haemorrhagic hepatic cyst
10067796	Haemorrhagic hepatic cyst
10019013	Haemorrhagic infarction
10089844	Haemorrhagic intestinal infarction
10089844	Haemorrhagic intestinal infarction
10076058	Haemorrhagic necrotic pancreatitis
10076058	Haemorrhagic necrotic pancreatitis
10085070	Haemorrhagic occlusive retinal vasculitis
10085070	Haemorrhagic occlusive retinal vasculitis
10085070	Haemorrhagic occlusive retinal vasculitis
10060781	Haemorrhagic ovarian cyst
10060781	Haemorrhagic ovarian cyst
10060781	Haemorrhagic ovarian cyst
10090638	Haemorrhagic pancreatic cyst
10090638	Haemorrhagic pancreatic cyst
10090638	Haemorrhagic pancreatic cyst
10090638	Haemorrhagic pancreatic cyst
10077933	Haemorrhagic pneumonia
10077933	Haemorrhagic pneumonia
10077933	Haemorrhagic pneumonia
10019016	Haemorrhagic stroke
10019016	Haemorrhagic stroke

10072256	Haemorrhagic thyroid cyst
10072256	Haemorrhagic thyroid cyst
10072256	Haemorrhagic thyroid cyst
10055677	Haemorrhagic transformation stroke
10055677	Haemorrhagic transformation stroke
10054096	Haemorrhagic tumour necrosis
10054096	Haemorrhagic tumour necrosis
10059499	Haemorrhagic urticaria
10059499	Haemorrhagic urticaria
10071252	Haemorrhagic vasculitis
10071252	Haemorrhagic vasculitis
10071252	Haemorrhagic vasculitis
10071252	Haemorrhagic vasculitis
10085423	Heavy menstrual bleeding
10019883	Hereditary haemorrhagic telangiectasia
10019883	Hereditary haemorrhagic telangiectasia
10019883	Hereditary haemorrhagic telangiectasia
10019883	Hereditary haemorrhagic telangiectasia
10022559	Intermenstrual bleeding
10048891	Nail bed bleeding
10048891	Nail bed bleeding
10048891	Nail bed bleeding
10029132	Nephritis haemorrhagic

10029132	Nephritis haemorrhagic
10030219	Oesophagitis haemorrhagic
10030219	Oesophagitis haemorrhagic
10033650	Pancreatitis haemorrhagic
10086195	Peripheral exudative haemorrhagic chorioretinopathy
10086195	Peripheral exudative haemorrhagic chorioretinopathy
10079859	Posthaemorrhagic hydrocephalus
10079859	Posthaemorrhagic hydrocephalus
10036778	Proctitis haemorrhagic
10036778	Proctitis haemorrhagic
10051447	Retinopathy haemorrhagic
10051447	Retinopathy haemorrhagic
10051447	Retinopathy haemorrhagic
10051447	Retinopathy haemorrhagic
10049771	Shock haemorrhagic
10049771	Shock haemorrhagic
10060712	Skin neoplasm bleeding
10060712	Skin neoplasm bleeding
10060712	Skin neoplasm bleeding
10042132	Stomatitis haemorrhagic
10042132	Stomatitis haemorrhagic

10088030	Unexpected vaginal bleeding on hormonal IUD
10088030	Unexpected vaginal bleeding on hormonal IUD
10088030	Unexpected vaginal bleeding on hormonal IUD
10047998	Withdrawal bleed
10047998	Withdrawal bleed
10067788	Abdominal wall haemorrhage
10067788	Abdominal wall haemorrhage
10067788	Abdominal wall haemorrhage
10075101	Administration site haemorrhage
10075101	Administration site haemorrhage
10075101	Administration site haemorrhage
10001361	Adrenal haemorrhage
10001361	Adrenal haemorrhage
10001361	Adrenal haemorrhage
10072601	Amyloid related imaging abnormality-microhaemorrhages and haemosiderin deposits
10072601	Amyloid related imaging abnormality-microhaemorrhages and haemosiderin deposits
10079765	Anal fissure haemorrhage
10079765	Anal fissure haemorrhage
10049555	Anal haemorrhage
10049555	Anal haemorrhage
10063896	Anal ulcer haemorrhage

10063896	Anal ulcer haemorrhage
10056346	Anastomotic haemorrhage
10056346	Anastomotic haemorrhage
10002244	Anastomotic ulcer haemorrhage
10002244	Anastomotic ulcer haemorrhage
10002244	Anastomotic ulcer haemorrhage
10068925	Anorectal varices haemorrhage
10068925	Anorectal varices haemorrhage
10068925	Anorectal varices haemorrhage
10072694	Application site haemorrhage
10072694	Application site haemorrhage
10072694	Application site haemorrhage
10060964	Arterial haemorrhage
10055123	Arteriovenous fistula site haemorrhage
10055123	Arteriovenous fistula site haemorrhage
10055126	Arteriovenous graft site haemorrhage
10055126	Arteriovenous graft site haemorrhage
10067057	Basal ganglia haemorrhage
10067057	Basal ganglia haemorrhage
10073581	Bone marrow haemorrhage
10073581	Bone marrow haemorrhage
10006145	Brain stem haemorrhage
10006145	Brain stem haemorrhage

10071205	Brain stem microhaemorrhage
10071205	Brain stem microhaemorrhage
10006254	Breast haemorrhage
10006254	Breast haemorrhage
10065739	Bronchial haemorrhage
10065739	Bronchial haemorrhage
10079163	Bronchial varices haemorrhage
10079163	Bronchial varices haemorrhage
10051099	Catheter site haemorrhage
10051099	Catheter site haemorrhage
10051099	Catheter site haemorrhage
10072043	Central nervous system haemorrhage
10072043	Central nervous system haemorrhage
10008030	Cerebellar haemorrhage
10008030	Cerebellar haemorrhage
10071206	Cerebellar microhaemorrhage
10071206	Cerebellar microhaemorrhage
10082099	Cerebral cyst haemorrhage
10082099	Cerebral cyst haemorrhage
10082099	Cerebral cyst haemorrhage
10008111	Cerebral haemorrhage
10008111	Cerebral haemorrhage
10050157	Cerebral haemorrhage foetal

10050157	Cerebral haemorrhage foetal
10050157	Cerebral haemorrhage foetal
10008112	Cerebral haemorrhage neonatal
10008112	Cerebral haemorrhage neonatal
10008112	Cerebral haemorrhage neonatal
10067277	Cerebral microhaemorrhage
10067277	Cerebral microhaemorrhage
10050022	Cervix haemorrhage uterine
10050022	Cervix haemorrhage uterine
10008786	Choroidal haemorrhage
10008786	Choroidal haemorrhage
10057417	Ciliary body haemorrhage
10057417	Ciliary body haemorrhage
10010719	Conjunctival haemorrhage
10010719	Conjunctival haemorrhage
10013839	Duodenal ulcer haemorrhage
10013839	Duodenal ulcer haemorrhage
10014009	Ear haemorrhage
10014009	Ear haemorrhage
10014009	Ear haemorrhage
10073681	Epidural haemorrhage
10073681	Epidural haemorrhage
10073681	Epidural haemorrhage

10078254	Extra-axial haemorrhage
10078254	Extra-axial haemorrhage
10078254	Extra-axial haemorrhage
10015926	Eye haemorrhage
10015926	Eye haemorrhage
10015926	Eye haemorrhage
10016871	Foetal-maternal haemorrhage
10016871	Foetal-maternal haemorrhage
10017788	Gastric haemorrhage
10017788	Gastric haemorrhage
10017826	Gastric ulcer haemorrhage
10017826	Gastric ulcer haemorrhage
10017829	Gastric ulcer haemorrhage, obstructive
10017829	Gastric ulcer haemorrhage, obstructive
10057572	Gastric varices haemorrhage
10057572	Gastric varices haemorrhage
10057572	Gastric varices haemorrhage
10053768	Gastroduodenal haemorrhage
10053768	Gastroduodenal haemorrhage
10085369	Gastrointestinal anastomotic haemorrhage
10085369	Gastrointestinal anastomotic haemorrhage
10085369	Gastrointestinal anastomotic haemorrhage
10017955	Gastrointestinal haemorrhage

10017955	Gastrointestinal haemorrhage
10074437	Gastrointestinal polyp haemorrhage
10074437	Gastrointestinal polyp haemorrhage
10074437	Gastrointestinal polyp haemorrhage
10056743	Gastrointestinal ulcer haemorrhage
10056743	Gastrointestinal ulcer haemorrhage
10088347	Gastrooesophageal haemorrhage
10088347	Gastrooesophageal haemorrhage
10066597	Gastrooesophageal variceal haemorrhage prophylaxis
10061178	Genital haemorrhage
10061178	Genital haemorrhage
10063577	Graft haemorrhage
10063577	Graft haemorrhage
10055798	Haemorrhage
10055803	Haemorrhage coronary artery
10055803	Haemorrhage coronary artery
10061191	Haemorrhage foetal
10061191	Haemorrhage foetal
10018981	Haemorrhage in pregnancy
10018981	Haemorrhage in pregnancy
10018985	Haemorrhage intracranial
10018985	Haemorrhage intracranial
10061993	Haemorrhage neonatal

10061993	Haemorrhage neonatal
10018999	Haemorrhage subcutaneous
10018999	Haemorrhage subcutaneous
10019001	Haemorrhage subepidermal
10019001	Haemorrhage subepidermal
10055847	Haemorrhage urinary tract
10055847	Haemorrhage urinary tract
10054787	Haemorrhoidal haemorrhage
10054787	Haemorrhoidal haemorrhage
10087132	Hepatic artery haemorrhage
10087132	Hepatic artery haemorrhage
10087132	Hepatic artery haemorrhage
10019677	Hepatic haemorrhage
10019677	Hepatic haemorrhage
10019677	Hepatic haemorrhage
10053995	Implant site haemorrhage
10053995	Implant site haemorrhage
10053995	Implant site haemorrhage
10051100	Incision site haemorrhage
10051100	Incision site haemorrhage
10052844	Induced abortion haemorrhage
10052844	Induced abortion haemorrhage
10065464	Infusion site haemorrhage

10065464	Infusion site haemorrhage
10065464	Infusion site haemorrhage
10022067	Injection site haemorrhage
10022067	Injection site haemorrhage
10022067	Injection site haemorrhage
10073610	Instillation site haemorrhage
10073610	Instillation site haemorrhage
10073610	Instillation site haemorrhage
10075192	Internal haemorrhage
10059175	Intestinal haemorrhage
10059175	Intestinal haemorrhage
10078058	Intestinal varices haemorrhage
10078058	Intestinal varices haemorrhage
10061249	Intra-abdominal haemorrhage
10061249	Intra-abdominal haemorrhage
10061249	Intra-abdominal haemorrhage
10086946	Intracranial haemorrhage neonatal
10086946	Intracranial haemorrhage neonatal
10086946	Intracranial haemorrhage neonatal
10022775	Intracranial tumour haemorrhage
10022775	Intracranial tumour haemorrhage
10022775	Intracranial tumour haemorrhage
10067703	Intrapartum haemorrhage

10067703	Intrapartum haemorrhage
10022840	Intraventricular haemorrhage
10022840	Intraventricular haemorrhage
10022841	Intraventricular haemorrhage neonatal
10022841	Intraventricular haemorrhage neonatal
10022841	Intraventricular haemorrhage neonatal
10057418	Iris haemorrhage
10057418	Iris haemorrhage
10077666	Joint microhaemorrhage
10077666	Joint microhaemorrhage
10077666	Joint microhaemorrhage
10085298	Jugular vein haemorrhage
10085298	Jugular vein haemorrhage
10069930	Lacrimal haemorrhage
10069930	Lacrimal haemorrhage
10052534	Large intestinal haemorrhage
10052534	Large intestinal haemorrhage
10061262	Large intestinal ulcer haemorrhage
10061262	Large intestinal ulcer haemorrhage
10065740	Laryngeal haemorrhage
10065740	Laryngeal haemorrhage
10065740	Laryngeal haemorrhage
10049297	Lip haemorrhage

10049297	Lip haemorrhage
10050953	Lower gastrointestinal haemorrhage
10050953	Lower gastrointestinal haemorrhage
10074270	Lymph node haemorrhage
10074270	Lymph node haemorrhage
10056343	Mediastinal haemorrhage
10056343	Mediastinal haemorrhage
10075578	Medical device site haemorrhage
10075578	Medical device site haemorrhage
10075578	Medical device site haemorrhage
10060717	Mesenteric haemorrhage
10060717	Mesenteric haemorrhage
10028024	Mouth haemorrhage
10028024	Mouth haemorrhage
10076048	Mucocutaneous haemorrhage
10076048	Mucocutaneous haemorrhage
10076048	Mucocutaneous haemorrhage
10061298	Mucosal haemorrhage
10061298	Mucosal haemorrhage
10028309	Muscle haemorrhage
10028309	Muscle haemorrhage
10028309	Muscle haemorrhage
10048849	Myocardial haemorrhage

10048849	Myocardial haemorrhage
10062955	Naevus haemorrhage
10062955	Naevus haemorrhage
10062955	Naevus haemorrhage
10074159	Neonatal gastrointestinal haemorrhage
10074159	Neonatal gastrointestinal haemorrhage
10074159	Neonatal gastrointestinal haemorrhage
10057571	Ocular retrobulbar haemorrhage
10057571	Ocular retrobulbar haemorrhage
10057571	Ocular retrobulbar haemorrhage
10030172	Oesophageal haemorrhage
10030172	Oesophageal haemorrhage
10030202	Oesophageal ulcer haemorrhage
10030202	Oesophageal ulcer haemorrhage
10030210	Oesophageal varices haemorrhage
10030210	Oesophageal varices haemorrhage
10030210	Oesophageal varices haemorrhage
10085045	Omental haemorrhage
10085045	Omental haemorrhage
10085045	Omental haemorrhage
10030919	Optic disc haemorrhage
10030919	Optic disc haemorrhage
10030941	Optic nerve sheath haemorrhage

10030941	Optic nerve sheath haemorrhage
10031045	Orbital haemorrhage
10031045	Orbital haemorrhage
10031045	Orbital haemorrhage
10065741	Ovarian haemorrhage
10065741	Ovarian haemorrhage
10033625	Pancreatic haemorrhage
10033625	Pancreatic haemorrhage
10083813	Pancreatic pseudocyst haemorrhage
10083813	Pancreatic pseudocyst haemorrhage
10059164	Papillary muscle haemorrhage
10059164	Papillary muscle haemorrhage
10080108	Paranasal sinus haemorrhage
10080108	Paranasal sinus haemorrhage
10059051	Parathyroid haemorrhage
10059051	Parathyroid haemorrhage
10051166	Parotid gland haemorrhage
10051166	Parotid gland haemorrhage
10063678	Pelvic haemorrhage
10063678	Pelvic haemorrhage
10063678	Pelvic haemorrhage
10034305	Penile haemorrhage
10034305	Penile haemorrhage

10034344	Peptic ulcer haemorrhage
10034344	Peptic ulcer haemorrhage
10034476	Pericardial haemorrhage
10034476	Pericardial haemorrhage
10071697	Periorbital haemorrhage
10071697	Periorbital haemorrhage
10071697	Periorbital haemorrhage
10071697	Periorbital haemorrhage
10072693	Peripartum haemorrhage
10072693	Peripartum haemorrhage
10076706	Periventricular haemorrhage neonatal
10076706	Periventricular haemorrhage neonatal
10076706	Periventricular haemorrhage neonatal
10034827	Pharyngeal haemorrhage
10034827	Pharyngeal haemorrhage
10049760	Pituitary haemorrhage
10049760	Pituitary haemorrhage
10049760	Pituitary haemorrhage
10035121	Placenta praevia haemorrhage
10035121	Placenta praevia haemorrhage
10036246	Post abortion haemorrhage
10036246	Post abortion haemorrhage
10051077	Post procedural haemorrhage

10051077	Post procedural haemorrhage
10071639	Post-traumatic punctate intraepidermal haemorrhage
10071639	Post-traumatic punctate intraepidermal haemorrhage
10071639	Post-traumatic punctate intraepidermal haemorrhage
10055870	Postmenopausal haemorrhage
10055870	Postmenopausal haemorrhage
10036417	Postpartum haemorrhage
10036417	Postpartum haemorrhage
10071229	Procedural haemorrhage
10071229	Procedural haemorrhage
10036960	Prostatic haemorrhage
10036960	Prostatic haemorrhage
10037313	Pulmonary alveolar haemorrhage
10037313	Pulmonary alveolar haemorrhage
10037313	Pulmonary alveolar haemorrhage
10037394	Pulmonary haemorrhage
10037394	Pulmonary haemorrhage
10082194	Pulmonary haemorrhage neonatal
10082194	Pulmonary haemorrhage neonatal
10082194	Pulmonary haemorrhage neonatal
10051101	Puncture site haemorrhage
10051101	Puncture site haemorrhage
10051101	Puncture site haemorrhage

10058940	Putamen haemorrhage
10058940	Putamen haemorrhage
10072281	Radiation associated haemorrhage
10072281	Radiation associated haemorrhage
10038063	Rectal haemorrhage
10038063	Rectal haemorrhage
10038081	Rectal ulcer haemorrhage
10038081	Rectal ulcer haemorrhage
10059846	Renal cyst haemorrhage
10059846	Renal cyst haemorrhage
10059846	Renal cyst haemorrhage
10038460	Renal haemorrhage
10038460	Renal haemorrhage
10038460	Renal haemorrhage
10038727	Respiratory tract haemorrhage
10038727	Respiratory tract haemorrhage
10038728	Respiratory tract haemorrhage neonatal
10038728	Respiratory tract haemorrhage neonatal
10038728	Respiratory tract haemorrhage neonatal
10038867	Retinal haemorrhage
10038867	Retinal haemorrhage
10038980	Retroperitoneal haemorrhage
10038980	Retroperitoneal haemorrhage

10038980	Retroperitoneal haemorrhage
10050508	Scleral haemorrhage
10050508	Scleral haemorrhage
10061361	Scrotal haemorrhage
10061361	Scrotal haemorrhage
10064265	Skin haemorrhage
10064265	Skin haemorrhage
10050377	Skin ulcer haemorrhage
10050377	Skin ulcer haemorrhage
10052535	Small intestinal haemorrhage
10052535	Small intestinal haemorrhage
10061550	Small intestinal ulcer haemorrhage
10061550	Small intestinal ulcer haemorrhage
10051297	Soft tissue haemorrhage
10051297	Soft tissue haemorrhage
10065742	Spermatic cord haemorrhage
10065742	Spermatic cord haemorrhage
10048992	Spinal cord haemorrhage
10048992	Spinal cord haemorrhage
10048992	Spinal cord haemorrhage
10049236	Spinal epidural haemorrhage
10049236	Spinal epidural haemorrhage
10049236	Spinal epidural haemorrhage

10073564	Spinal subarachnoid haemorrhage
10073564	Spinal subarachnoid haemorrhage
10073564	Spinal subarachnoid haemorrhage
10073563	Spinal subdural haemorrhage
10073563	Spinal subdural haemorrhage
10073563	Spinal subdural haemorrhage
10041647	Splenic haemorrhage
10041647	Splenic haemorrhage
10041647	Splenic haemorrhage
10068662	Splenic varices haemorrhage
10068662	Splenic varices haemorrhage
10068662	Splenic varices haemorrhage
10041663	Splinter haemorrhages
10041663	Splinter haemorrhages
10074557	Spontaneous haemorrhage
10074557	Spontaneous haemorrhage
10074508	Stoma site haemorrhage
10074508	Stoma site haemorrhage
10085612	Stress ulcer haemorrhage
10085612	Stress ulcer haemorrhage
10042316	Subarachnoid haemorrhage
10042316	Subarachnoid haemorrhage
10042316	Subarachnoid haemorrhage

10042317	Subarachnoid haemorrhage neonatal
10042317	Subarachnoid haemorrhage neonatal
10042317	Subarachnoid haemorrhage neonatal
10042317	Subarachnoid haemorrhage neonatal
10071010	Subchorionic haemorrhage
10071010	Subchorionic haemorrhage
10042364	Subdural haemorrhage
10042364	Subdural haemorrhage
10042364	Subdural haemorrhage
10042365	Subdural haemorrhage neonatal
10042365	Subdural haemorrhage neonatal
10042365	Subdural haemorrhage neonatal
10082459	Subendocardial haemorrhage
10082459	Subendocardial haemorrhage
10080900	Subgaleal haemorrhage
10080900	Subgaleal haemorrhage
10080900	Subgaleal haemorrhage
10051877	Testicular haemorrhage
10051877	Testicular haemorrhage
10090985	Thalamic microhaemorrhage
10090985	Thalamic microhaemorrhage
10058939	Thalamus haemorrhage
10058939	Thalamus haemorrhage

10043449	Third stage postpartum haemorrhage
10043449	Third stage postpartum haemorrhage
10062744	Thoracic haemorrhage
10062744	Thoracic haemorrhage
10064224	Thyroid haemorrhage
10064224	Thyroid haemorrhage
10049870	Tongue haemorrhage
10049870	Tongue haemorrhage
10057450	Tonsillar haemorrhage
10057450	Tonsillar haemorrhage
10072228	Tooth pulp haemorrhage
10072228	Tooth pulp haemorrhage
10064946	Tooth socket haemorrhage
10064946	Tooth socket haemorrhage
10062543	Tracheal haemorrhage
10062543	Tracheal haemorrhage
10062543	Tracheal haemorrhage
10053476	Traumatic haemorrhage
10053476	Traumatic haemorrhage
10061387	Traumatic intracranial haemorrhage
10061387	Traumatic intracranial haemorrhage
10061387	Traumatic intracranial haemorrhage
10090895	Traumatic subarachnoid haemorrhage

10090895	Traumatic subarachnoid haemorrhage
10049750	Tumour haemorrhage
10049750	Tumour haemorrhage
10061577	Ulcer haemorrhage
10061577	Ulcer haemorrhage
10064534	Umbilical cord haemorrhage
10064534	Umbilical cord haemorrhage
10064534	Umbilical cord haemorrhage
10045455	Umbilical haemorrhage
10045455	Umbilical haemorrhage
10046274	Upper gastrointestinal haemorrhage
10046274	Upper gastrointestinal haemorrhage
10065743	Ureteric haemorrhage
10065743	Ureteric haemorrhage
10049710	Urethral haemorrhage
10049710	Urethral haemorrhage
10046528	Urinary bladder haemorrhage
10046528	Urinary bladder haemorrhage
10050058	Urogenital haemorrhage
10050058	Urogenital haemorrhage
10050058	Urogenital haemorrhage
10046788	Uterine haemorrhage
10046788	Uterine haemorrhage

10069475	Vaccination site haemorrhage
10069475	Vaccination site haemorrhage
10069475	Vaccination site haemorrhage
10046910	Vaginal haemorrhage
10046910	Vaginal haemorrhage
10077643	Vascular access site haemorrhage
10077643	Vascular access site haemorrhage
10077643	Vascular access site haemorrhage
10084092	Vascular anastomotic haemorrhage
10084092	Vascular anastomotic haemorrhage
10077721	Vascular graft haemorrhage
10077721	Vascular graft haemorrhage
10065441	Venous haemorrhage
10054092	Vessel puncture site haemorrhage
10054092	Vessel puncture site haemorrhage
10054092	Vessel puncture site haemorrhage
10047655	Vitreous haemorrhage
10047655	Vitreous haemorrhage
10088766	Vocal cord haemorrhage
10088766	Vocal cord haemorrhage
10088766	Vocal cord haemorrhage
10063816	Vulval haemorrhage
10063816	Vulval haemorrhage

10051373	Wound haemorrhage
10051373	Wound haemorrhage

Supplementary Table S2. Number of reports pertaining to combined anticoagulant and antiplatelet drugs.

Anticoagulant and antiplatelet combinations	Number of reports at each stage		
	**Screening**	**Identification**	**Included in the final analysis**
**Acenocoumarol combinations**			
Aspirin	1471	240	207
Cilostazol	9	1	1
Clopidogrel	922	158	130
Dipyridamole	31	4	3
Prasugrel	6	1	1
Ticagrelor	77	11	10
**Apixaban combinations**			
Aspirin	30038	3843	3334
Cilostazol	299	49	44
Clopidogrel	17529	2460	2168
Dipyridamole	209	14	12
Prasugrel	372	49	40
Ticagrelor	892	127	122
**Betrixaban combinations**			
Aspirin	No reports		
Cilostazol			
Clopidogrel			
Dipyridamole			
Prasugrel			
Ticagrelor			
**Dabigatran combinations**			
Aspirin	20423	1911	1533
Cilostazol	415	36	29
Clopidogrel	8012	824	675
Dipyridamole	499	36	28
Prasugrel	268	21	18
Ticagrelor	243	33	29
**Edoxaban combinations**			
Aspirin	887	86	77
Cilostazol	No reports		
Clopidogrel	742	90	83
Dipyridamole	No reports		

Prasugrel	189	21	17
Ticagrelor	40	3	3
**Rivaroxaban combinations**			
Aspirin	108559	15134	12801
Cilostazol	1089	106	89
Clopidogrel	30466	4003	3431
Dipyridamole	552	41	36
Prasugrel	947	123	107
Ticagrelor	1817	242	212
**Warfarin combinations**			
Aspirin	35994	4407	3841
Cilostazol	899	100	84
Clopidogrel	19084	2541	2140
Dipyridamole	1432	188	155
Prasugrel	498	97	83
Ticagrelor	1054	127	110

Supplementary Table S3. Demographic characteristics in the reports related to hemorrhage with acenocoumarol.

Variables		Values (n=56)
Age group [n (%)]	<18 years	0
	≥18-<44 years	1(1.7)
	≥45-<64 years	8 (14)
	≥65 years	40 (70.1)
	Not reported	8 (14)
Mean (SD) age (years)		74.4 (27.3)
Median (range) age (years)		77 (41-93)
Gender distribution [n (%)]	Male	30 (53.5)
	Female	24 (42)
	Unknown	2 (3.5)
Year of receiving the report [n (%)]	2004-2008	1 (1.7)
	2009-2012	4 (7.1)
	2013-2016	40 (71)
	2017-2020	10 (17)
	2021-2024	1 (1.7)
Top reporting countries [n (%)]	ES	28 (50)
	FR	6 (10)
	IT	6 (10)
	NL	6 (10)
	CH	2 (3.5)

ES: Estonia; FR: France; IT: Italy; NL: Netherlands; CH: China.

Supplementary Table S4. Demographic characteristics in the reports related to hemorrhage with apixaban.

**Variables**		**Values (n= 21255)**
Age group [n (%)]	<18 years	21(0.03)
	≥18-<45 years	296(1.4)
	≥45-<65 years	1678(7.8)
	≥65 years	11558(54.3)
	Not reported	7701(36.2)
Mean (SD) age (years)		75.8(37.7)
Median (range) age (years)		78(0-123)
Gender distribution [n (%)]	Male	9770(45.9)
	Female	9080(42.7)
	Unknown	2324(10.9)
Year of receiving the report [n (%)]	2004-2008	0
	2009-2012	1(0.004)
	2013-2016	4230(19.9)
	2017-2020	9986(46.9)
	2021-2024	7038(33.1)
Top reporting countries [n (%)]	USA	1418(6.6)
	Canada	338(1.7)
	UK	733(3.4)
	France	726(3.4)
	Japan	1545(7.2)
	Others and not reported	16495(77.6)

USA: The United States of America; UK: The United Kingdom.

Supplementary Table S5. Demographic characteristics in the reports related to hemorrhage with dabigatran.

**Variables**		**Values (n=14618)**
Age group [n (%)]	<18 years	11(0.07)
	≥18-<44 years	87(0.5)
	≥45-<64 years	987(6.7)
	≥65 years	7960(54.4)
	Not reported	5571(38.1)
Mean (SD) age (years)		76.39(10.6)
Median (range) age (years)		78(0-105)
Gender distribution [n (%)]	Male	6350(43.4)
	Female	6471(44.2)
	Unknown	2247(15)
Year of receiving the report [n (%)]	2004-2008	0
	2009-2012	6183(42.2)
	2013-2016	2135(14.6)
	2017-2020	5547(37.9)
	2021-2024	763(5.2)
Top reporting countries [n (%)]	USA	5701(38.9)
	Canada	124(0.8)
	UK	40(0.2)
	France	385(2.6)
	Japan	23(0.1)
	Others and not reported	8345(57)

USA: The United States of America; UK: The United Kingdom.

Supplementary Table S6. Demographic characteristics in the reports related to hemorrhage with edoxaban.

Variables		Values (n= 332)
Age group [n (%)]	<18 years	0
	≥18-<45 years	6(1.8)
	≥45-<65 years	25(7.5)
	≥65 years	206(62)
	Not reported	95(28.6)
Mean (SD) age (years)		76(11.5)
Median (range) age (years)		79(29-97)
Gender distribution [n (%)]	Male	147(44.2)
	Female	109(32.8)
	Unknown	79(23.7)
Year of receiving the report [n (%)]	2004-2008	0
	2009-2012	0
	2013-2016	19(5.7)
	2017-2020	85(25.6)
	2021-2024	228(68.6)
Top reporting countries [n (%)]	USA	26(7.8)
	Canada	30(0.9)
	UK	38(11)
	Japan	5(1.5)
	Others and not reported	233(70.26)

USA: The United States of America; UK: The United Kingdom.

Supplementary Table S7. Demographic characteristics in the reports related to hemorrhage with rivaroxaban.

Variables		Values (n= 40727)
Age group [n (%)]	<18 years	26(0.06)
	≥18-<45 years	1207(2.9)
	≥45-<65 years	4992(12.2)
	≥65 years	16492(40.4)
	Not reported	18010(44.2)
Mean (SD) age (years)		70.47(13.7)
Median (range) age (years)		73(0-114)
Gender distribution [n (%)]	Male	16326(40)
	Female	15786(38.7)
	Unknown	8597(21)
Year of receiving the report [n (%)]	2004-2008	0
	2009-2012	1123(2.7)
	2013-2016	12420(30.4)
	2017-2020	4937(12.1)
	2021-2024	2917(7.1)
Top reporting countries [n (%)]	USA	34326(84)
	Canada	429(1)
	UK	19(0.04)
	France	32(0.07)
	Japan	625(1.5)
	Others and not reported	5296(13)

USA: The United States of America; UK: The United Kingdom.

Supplementary Table S8. Demographic characteristics in the reports related to hemorrhage with warfarin.

Variables		Values (n=19400)
Age group [n (%)]	<18 years	125(.64)
	≥18-<45 years	700(3.6)
	≥45-<65 years	3142(16)
	≥65 years	10242(52.7)
	Not reported	5191(26.7)
Mean (SD) age (years)		63(14.8)
Median (range) age (years)		73(0-103)
Gender distribution [n (%)]	Male	10819(26.5)
	Female	6327(15.5)
	Unknown	2547(6.2)
Year of receiving the report [n (%)]	2004-2008	3583(8.7)
	2009-2012	7256(17.8)
	2013-2016	5844(14.3)
	2017-2020	4691(11.5)
	2021-2024	1609(3.9)
Top reporting countries [n (%)]	USA	13821(33.9)
	Canada	173(0.4)
	UK	990(2.4)
	France	588(1.4)
	Japan	223(0.5)
	Others and not reported	3595(8.8)

USA: The United States of America; UK: The United Kingdom.

Supplementary Table S9. Demographic characteristics in the reports related to hemorrhage with aspirin.

Variables		Values (n=12889)
Age group [n (%)]	<18 years	117(0.9)
	≥18-<45 years	489(3.7)
	≥45-<65 years	2614(20.2)
	≥65 years	8261(64)
	Not reported	1408(10.9)
Mean (SD) age (years)		69.73(14.3)
Median (range) age (years)		72(0-104)
Gender distribution [n (%)]	Male	7087(54.9)
	Female	5026(38.9)
	Unknown	920(7.1)
Year of receiving the report [n (%)]	2004-2008	67(0.5)
	2009-2012	154(1.1)
	2013-2016	4534(35.1)
	2017-2020	6585(51)
	2021-2024	1549(12)
Top reporting countries [n (%)]	USA	9081(70.4)
	Canada	91(0.7)
	UK	434(3.3)
	France	65(0.5)
	Japan	540(4.1)
	Others and not reported	2679(20.7)

USA: The United States of America; UK: The United Kingdom.

Supplementary Table S10. Demographic characteristics in the reports related to hemorrhage with cilostazol.

**Variables**		**Values (n=324)**
Age group [n (%)]	<18 years	0
	≥18-<45 years	8(2.4)
	≥45-<65 years	51(15.7)
	≥65 years	99(30.5)
	Not reported	71(21.9)
Mean (SD) age (years)		72.6(11.4)
Median (range) age (years)		76(35-97)
Gender distribution [n (%)]	Male	181(55.8)
	Female	90(27.7)
	Unknown	51(15.7)
Year of receiving the report [n (%)]	2004-2008	84(25.9)
	2009-2012	106(32.7)
	2013-2016	55(16.9)
	2017-2020	66(20.3)
	2021-2024	13(4)
Top reporting countries [n (%)]	USA	15(4)
	UK	11(3.3)
	Japan	230(70.9)
	Others and not reported	68(20.9)

USA: The United States of America; UK: The United Kingdom.

Supplementary Table S11. Demographic characteristics in the reports related to hemorrhage with clopidogrel.

Variables		Values (n= 13968)
Age group [n (%)]	<18 years	21(0.15)
	≥18-<45 years	234(1.6)
	≥45-<65 years	2160(15.4)
	≥65 years	6817(48.8)
	Not reported	4737(33.9)
Mean (SD) age (years)		71.69(12.6)
Median (range) age (years)		73(0-103)
Gender distribution [n (%)]	Male	6662(47.6)
	Female	4959(35.5)
	Unknown	2311(16.5)
Year of receiving the report [n (%)]	2004-2008	1221(8.7)
	2009-2012	930(6.6)
	2013-2016	4821(34.5)
	2017-2020	4114(29.4)
	2021-2024	2883(20.6)
Top reporting countries [n (%)]	USA	6521(46.6)
	Canada	24(0.1)
	UK	1299(9.2)
	France	542(3.8)
	Japan	327(2.3)
	Others and not reported	5255(37.6)

USA: The United States of America; UK: The United Kingdom.

Supplementary Table S12. Demographic characteristics in the reports related to hemorrhage with dipyridamole.

Variables		Values (n= 517)
Age group [n (%)]	<18 years	28(5.4)
	≥18-<45 years	6(1)
	≥45-<65 years	58(11.2)
	≥65 years	195(37.7)
	Not reported	230(44.4)
Mean (SD) age (years)		66.26(23.3)
Median (range) age (years)		71(1-101)
Gender distribution [n (%)]	Male	226(43.7)
	Female	224(43.3)
	Unknown	105(20.3)
Year of receiving the report [n (%)]	2004-2008	191(36.9)
	2009-2012	182(35.2)
	2013-2016	58(11.2)
	2017-2020	60(11.6)
	2021-2024	26(5.02)
Top reporting countries [n (%)]	USA	57(11)
	Canada	126(24.3)
	UK	18(3.4)
	France	6(1.1)
	Japan	13(2.5)
	Others and not reported	297(57.4)

USA: The United States of America; UK: The United Kingdom.

Supplementary Table S13. Demographic characteristics in the reports related to hemorrhage with prasugrel.

**Variables**		**Values (n=2408)**
Age group [n (%)]	<18 years	2(0.08)
	≥18-<45 years	69(2.8)
	≥45-<65 years	569(23.6)
	≥65 years	669(27.7)
	Not reported	739(30.6)
Mean (SD) age (years)		63.9(12)
Median (range) age (years)		65(5-100)
Gender distribution [n (%)]	Male	1269(52.6)
	Female	740(30.7)
	Unknown	68(2.8)
Year of receiving the report [n (%)]	2004-2008	0
	2009-2012	550(22.8)
	2013-2016	1147(47.6)
	2017-2020	207(8.5)
	2021-2024	144(5.9)
Top reporting countries [n (%)]	USA	1541 (63.8)
	Canada	5(0.2)
	UK	14(0.58)
	France	35(1.4)
	Japan	189(7.8)
	Others and not reported	624(25.9)

USA: The United States of America; UK: The United Kingdom.

Supplementary Table S14. Demographic characteristics in the reports related to hemorrhage with ticagrelor.

Variables		Values (n= 2923)
Age group [n (%)]	<18 years	3(0.1)
	≥18-<45 years	66(2.2)
	≥45-<65 years	610(20.8)
	≥65 years	1109(37.9)
	Not reported	1133(38.7)
Mean (SD) age (years)		67.41(12.06)
Median (range) age (years)		68(0-96)
Gender distribution [n (%)]	Male	1716(58.7)
	Female	992(33.9)
	Unknown	178(6.08)
Year of receiving the report [n (%)]	2004-2008	0
	2009-2012	180(6)
	2013-2016	1150(39.3)
	2017-2020	989(33.8)
	2021-2024	604(20.66)
Top reporting countries [n (%)]	USA	1478(50.5)
	Canada	39(1.3)
	UK	113(3.8)
	France	107(3.6)
	Japan	15(0.5)
	Others and not reported	1171(40)

USA: The United States of America; UK: The United Kingdom.

Supplementary Table S15. Demographic characteristics in the reports related to hemorrhage with acenocoumarol with aspirin.

Variables		Values (n= 207)
Age group [n (%)]	<18 years	0
	≥18-<45 years	8 (4.6)
	≥45-<65 years	47 (26.5)
	≥65 years	120 (68.6)
	Not reported	32 (18.3)
Mean (SD) age (years)		69.6 (13.3)
Median (range) age (years)		74 (41-91)
Gender distribution [n (%)]	Male	134 (64.7)
	Female	49 (23.7)
	Unknown	24 (11.6)
Year of receiving the report [n (%)]	2004-2008	0
	2009-2012	4 (2.3)
	2013-2016	50 (28.6)
	2017-2020	112 (54.1)
	2021-2024	41 (19.8)
Top reporting countries [n (%)]	UK	13 (6.3)
	France	7 (3.4)
	Poland	4 (1.9)
	Switzerland	2 (0.95)
	Others and not reported	180 (87)

UK: The United Kingdom.

Supplementary Table S16. Demographic characteristics in the reports related to hemorrhage with acenocoumarol with cilostazol.

**Variables**		**Values (n= 9)**
Age group [n (%)]	≥65 years	9(100)
Mean (SD) age (years)		68
Median (range) age (years)		68
Gender distribution [n (%)]	Male	9(100)
Year of receiving the report [n (%)]	2021-2024	9(100)
Top reporting countries [n (%)]	Others and not reported	9(100)

Supplementary Table S17. Demographic characteristics in the reports related to hemorrhage with acenocoumarol with clopidogrel.

Variables		Values (n=130)
Age group [n (%)]	<18 years	0
	≥18-<45 years	6(4.6)
	≥45-<65 years	22(16.9)
	≥65 years	79(60.7)
	Not reported	23(17.6)
Mean (SD) age (years)		71.3(12.8)
Median (range) age (years)		76(41-91)
Gender distribution [n (%)]	Male	89(68.4)
	Female	24(18.4)
	Unknown	17(13)
Year of receiving the report [n (%)]	2004-2008	11(8.4)
	2009-2012	16(12.3)
	2013-2016	10(7.6)
	2017-2020	71(54.6)
	2021-2024	22(16.9)
Top reporting countries [n (%)]	UK	1(0.7)
	Others and not reported	129(99.2)

UK: The United Kingdom.

Supplementary Table S18. Demographic characteristics in the reports related to hemorrhage with acenocoumarol with dipyridamole.

**Variables**		**Values (n= 3)**
Age group [n (%)]	≥65 years	1(33.3)
	Not reported	2(66.6)
Mean (SD) age (years)		65
Median (range) age (years)		65
Gender distribution [n (%)]	Male	2(66.6)
	Female	0
	Unknown	1(33.3)
Year of receiving the report [n (%)]	2004-2008	1(33.3)
	2009-2012	1(33.3)
	2013-2016	1(33.3)
	2017-2020	0
	2021-2024	0
Top reporting countries [n (%)]	Others and not reported	3(100)

Supplementary Table S19. Demographic characteristics in the reports related to hemorrhage with acenocoumarol with prasugrel.

**Variables**		**Values (n=1)**
Age group [n (%)]	≥65 years	1(100)
Mean (SD) age (years)		72
Median (range) age (years)		72
Gender distribution [n (%)]	Male	1(100)
Year of receiving the report [n (%)]	2013-2016	1(100)
Top reporting countries [n (%)]	Others and not reported	1(100)

Supplementary Table S20. Demographic characteristics in the reports related to hemorrhage with acenocoumarol with ticagrelor.

**Variables**		**Values (n=10)**
Age group [n (%)]	<18 years	0
	≥18-<45 years	6(60)
	≥45-<65 years	0
	≥65 years	2(20)
	Not reported	2(20)
Mean (SD) age (years)		48.62(14.5)
Median (range) age (years)		41(41-78)
Gender distribution [n (%)]	Male	8(80)
	Female	0
	Unknown	2(20)
Year of receiving the report [n (%)]	2017-2020	10(100)
Top reporting countries [n (%)]	Others and not reported	10(100)

Supplementary Table S21. Demographic characteristics in the reports related to hemorrhage with apixaban with aspirin.

Variables		Values (n=3334)
Age group [n (%)]	<18 years	5(0.1)
	≥18-<45 years	27(0.8)
	≥45-<65 years	336(10)
	≥65 years	2268(68)
	Not reported	698(20.95)
Mean (SD) age (years)		74.9(11.19)
Median (range) age (years)		76(1-100)
Gender distribution [n (%)]	Male	1940(58.1)
	Female	1295(38.8)
	Unknown	257(7.7)
Year of receiving the report [n (%)]	2004-2008	0
	2009-2012	1(0.29)
	2013-2016	494(14.8)
	2017-2020	1562(46.8)
	2021-2024	1277(38.3)
Top reporting countries [n (%)]	USA	1976(59.2)
	Canada	116(3.4)
	UK	138(4.1)
	France	133(3.9)
	Japan	168(5)
	Others and not reported	803(24.08)

USA: The United States of America; UK: The United Kingdom.

Supplementary Table S22. Demographic characteristics in the reports related to hemorrhage with apixaban with cilostazol.

Variables		Values (n= 44)
Age group [n (%)]	<18 years	1(2.2)
	≥18-<45 years	0
	≥45-<65 years	2(4.4)
	≥65 years	35(79.5)
	Not reported	6(13.6)
Mean (SD) age (years)		77.0(15.38)
Median (range) age (years)		79.5(1-97)
Gender distribution [n (%)]	Male	34(77.27)
	Female	8(18.1)
	Unknown	2(4.5)
Year of receiving the report [n (%)]	2004-2008	0
	2009-2012	0
	2013-2016	12(27.2)
	2017-2020	18(40.9)
	2021-2024	14(31.8)
Top reporting countries [n (%)]	USA	12(27.2)
	UK	2(4.5)
	Japan	28(63.6)
	Others and not reported	2(4.5)

USA: The United States of America; UK: The United Kingdom.

Supplementary Table S23. Demographic characteristics in the reports related to hemorrhage with apixaban with clopidogrel.

Variables		Values (n=2168)
Age group [n (%)]	<18 years	2(0.09)
	≥18-<45 years	8(0.3)
	≥45-<65 years	219(10)
	≥65 years	1412(65.1)
	Not reported	526(24.2)
Mean (SD) age (years)		75.34(10.67)
Median (range) age (years)		77(1-100)
Gender distribution [n (%)]	Male	1190(54.8)
	Female	738(34)
	Unknown	235(10.8)
Year of receiving the report [n (%)]	2004-2008	0
	2009-2012	0
	2013-2016	255(11.76)
	2017-2020	1031(47.55)
	2021-2024	882(40.68)
Top reporting countries [n (%)]	USA	959(44.2)
	Canada	56(2.58)
	UK	201(9.2)
	France	114(5.2)
	Japan	108(4.9)
	Others and not reported	730(33.67)

USA: The United States of America; UK: The United Kingdom.

Supplementary Table S24. Demographic characteristics in the reports related to hemorrhage with apixaban with dipyridamole.

Variables		Values (n= 12)
Age group [n (%)]	<18 years	0
	≥18-<45 years	0
	≥45-<65 years	1(8.3)
	≥65 years	8(66.6)
	Not reported	3(25)
Mean (SD) age (years)		72.4(10.8)
Median (range) age (years)		70(52-83)
Gender distribution [n (%)]	Male	5(41.6)
	Female	4(33.3)
	Unknown	3(25)
Year of receiving the report [n (%)]	2004-2008	0
	2009-2012	0
	2013-2016	2(16.6)
	2017-2020	5(41.6)
	2021-2024	5(41.6)
Top reporting countries [n (%)]	USA	8(66.6)
	UK	1(8.3)
	Others and not reported	3(25)

USA: The United States of America; UK: The United Kingdom.

Supplementary Table S25. Demographic characteristics in the reports related to hemorrhage with apixaban with prasugrel.

Variables		Values (n= 40)
Age group [n (%)]	<18 years	0
	≥18-<45 years	1(2.5)
	≥45-<65 years	3(7.5)
	≥65 years	29(72.5)
	Not reported	7(17.5)
Mean (SD) age (years)		73.87(11.0)
Median (range) age (years)		75(32-90)
Gender distribution [n (%)]	Male	24(60)
	Female	12(30)
	Unknown	4(10)
Year of receiving the report [n (%)]	2004-2008	0
	2009-2012	0
	2013-2016	7(17.5)
	2017-2020	22(55)
	2021-2024	11(27.5)
Top reporting countries [n (%)]	USA	16(40)
	Canada	0
	UK	0
	France	1(2.5)
	Japan	22(55)

	Others and not reported	1(2.5)

USA: The United States of America; UK: The United Kingdom.

Supplementary Table S26. Demographic characteristics in the reports related to hemorrhage with apixaban with ticagrelor.

**Variables**		**Values (n= 122)**
Age group [n (%)]	<18 years	0
	≥18-<45 years	0
	≥45-<65 years	13(10.65)
	≥65 years	81(66.3)
	Not reported	28(22.95)
Mean (SD) age (years)		74.6(8.7)
Median (range) age (years)		76(49-92)
Gender distribution [n (%)]	Male	65(53.27)
	Female	44(36)
	Unknown	17(13.93)
Year of receiving the report [n (%)]	2004-2008	0
	2009-2012	0
	2013-2016	7(5.7)
	2017-2020	69(56.55)
	2021-2024	46(37.7)
Top reporting countries [n (%)]	USA	83(68)
	Canada	11(9)
	UK	12(9.8)
	France	3(2.4)
	Others and not reported	13(10.65)

USA: The United States of America; UK: The United Kingdom.

Supplementary Table S27. Demographic characteristics in the reports related to hemorrhage with dabigatran with aspirin.

Variables		Values (n= 1533)
Age group [n (%)]	<18 years	3(0.19)
	≥18-<45 years	27(1.7)
	≥45-<65 years	173(11.2)
	≥65 years	1090(71.1)
	Not reported	240(15.65)
Mean (SD) age (years)		74.2(11.1)
Median (range) age (years)		75(12-96)
Gender distribution [n (%)]	Male	951(62)
	Female	585(38.1)
	Unknown	53(3.4)
Year of receiving the report [n (%)]	2004-2008	0
	2009-2012	86(5.6)
	2013-2016	274(17.87)
	2017-2020	1032(67.3)
	2021-2024	141(9.1)
Top reporting countries [n (%)]	USA	1106(72.1)
	Canada	24(1.5)
	Japan	15(0.97)
	Others and not reported	388(25.3)

USA: The United States of America.

Supplementary Table S28. Demographic characteristics in the reports related to hemorrhage with dabigatran with cilostazol.

Variables		Values (n=29)
Age group [n (%)]	<18 years	0
	≥18-<45 years	0
	≥45-<65 years	2(6.8)
	≥65 years	22(75.8)
	Not reported	5(17.2)
Mean (SD) age (years)		73.7(6.5)
Median (range) age (years)		72(63-89)
Gender distribution [n (%)]	Male	15(51.7)
	Female	14(48.2)
	Unknown	0
Year of receiving the report [n (%)]	2004-2008	0
	2009-2012	7(24.1)
	2013-2016	6(20.6)
	2017-2020	16(55.1)
	2021-2024	0
Top reporting countries [n (%)]	USA	24(82.7)
	Japan	3(10.34)
	Others and not reported	2(6.8)

USA: The United States of America.

Supplementary Table S29. Demographic characteristics in the reports related to hemorrhage with dabigatran with clopidogrel.

**Variables**		**Values (n= 675)**
Age group [n (%)]	<18 years	0
	≥18-<45 years	3(0.4)
	≥45-<65 years	88(13.0)
	≥65 years	471(69.7)
	Not reported	113(16.7)
Mean (SD) age (years)		74.8(9.64)
Median (range) age (years)		77(25-97)
Gender distribution [n (%)]	Male	422(62.5)
	Female	236(34.9)
	Unknown	39(5.7)
Year of receiving the report [n (%)]	2004-2008	0
	2009-2012	152(22.51)
	2013-2016	74(10.96)
	2017-2020	364(53.9)
	2021-2024	85(12.59)
Top reporting countries [n (%)]	USA	390(57.7)
	Canada	16(2.3)
	UK	14(2)
	France	9(1.3)
	Japan	5(0.7)

	Others and not reported	241(35.7)

USA: The United States of America; UK: The United Kingdom.

Supplementary Table S30. Demographic characteristics in the reports related to hemorrhage with dabigatran with dipyridamole.

**Variables**		**Values (n=28)**
Age group [n (%)]	<18 years	0
	≥18-<45 years	0
	≥45-<65 years	1(3.7)
	≥65 years	27(96.4)
	Not reported	0
Mean (SD) age (years)		73.6(7.5)
Median (range) age (years)		72(52-87)
Gender distribution [n (%)]	Male	0
	Female	10(35.7)
	Unknown	17(60.74)
Year of receiving the report [n (%)]	2004-2008	1(3.7)
	2009-2012	2(7.4)
	2013-2016	0
	2017-2020	24(85.71)
	2021-2024	1(3.7)
Top reporting countries [n (%)]	USA	27(96.4)
	Others and not reported	1(3.7)

USA: The United States of America.

Supplementary Table S31. Demographic characteristics in the reports related to hemorrhage with dabigatran with prasugrel.

Variables		Values (n=18)
Age group [n (%)]	<18 years	0
	≥18-<45 years	0
	≥45-<65 years	4(22.2)
	≥65 years	11(61.1)
	Not reported	3(16.6)
Mean (SD) age (years)		70.2(11.7)
Median (range) age (years)		72(44-88)
Gender distribution [n (%)]	Male	13(72.2)
	Female	5(27.7)
	Unknown	0
Year of receiving the report [n (%)]	2004-2008	0
	2009-2012	8(44.4)
	2013-2016	2(11.1)
	2017-2020	8(44.4)
	2021-2024	0
Top reporting countries [n (%)]	USA	17(94.4)
	Others and not reported	1(5.5)

USA: The United States of America.

Supplementary Table S32. Demographic characteristics in the reports related to hemorrhage with dabigatran with ticagrelor.

Variables		Values (n=29)
Age group [n (%)]	<18 years	0
	≥18-<45 years	0
	≥45-<65 years	3(10.3)
	≥65 years	22(75.86)
	Not reported	4(13.7)
Mean (SD) age (years)		74.84(7.1)
Median (range) age (years)		75(62-89)
Gender distribution [n (%)]	Male	22(75.8)
	Female	6(20.6)
	Unknown	1(3.4)
Year of receiving the report [n (%)]	2004-2008	0
	2009-2012	1(3.4)
	2013-2016	5(17.2)
	2017-2020	19(65.5)
	2021-2024	4(13.7)
Top reporting countries [n (%)]	USA	10(34.4)
	Canada	4(13.7)
	UK	1(3.4)
	France	4(13.7)

	Others and not reported	10(34.4)

USA: The United States of America; UK: The United Kingdom.

Supplementary Table S33. Demographic characteristics in the reports related to hemorrhage with edoxaban with aspirin.

**Variables**		**Values (n=77)**
Age group [n (%)]	<18 years	0
	≥18-<45 years	0
	≥45-<65 years	2(2.5)
	≥65 years	60(77.9)
	Not reported	15(19.4)
Mean (SD) age (years)		76.46(6.4)
Median (range) age (years)		75(61-88)
Gender distribution [n (%)]	Male	51(66.2)
	Female	18(23.3)
	Unknown	8(10.3)
Year of receiving the report [n (%)]	2004-2008	0
	2009-2012	0
	2013-2016	5(6.4)
	2017-2020	30(38.9)
	2021-2024	42(54.5)
Top reporting countries [n (%)]	USA	6(7.7)
	UK	2(2.5)
	Japan	21(27.2)

	Others and not reported	48(62.3)

USA: The United States of America; UK: The United Kingdom.

Supplementary Table S34. Demographic characteristics in the reports related to hemorrhage with edoxaban with clopidogrel.

Variables		Values (n=83)
Age group [n (%)]	<18 years	0
	≥18-<45 years	0
	≥45-<65 years	8(9.6)
	≥65 years	59(71.0)
	Not reported	16(19.2)
Mean (SD) age (years)		75.22(9.4)
Median (range) age (years)		75(54-90)
Gender distribution [n (%)]	Male	54(65.0)
	Female	15(18.0)
	Unknown	14(16.8)
Year of receiving the report [n (%)]	2004-2008	0
	2009-2012	0
	2013-2016	1(1.2)
	2017-2020	25(30.1)
	2021-2024	57(68.6)
Top reporting countries [n (%)]	USA	2(2.4)
	Canada	2(2.4)
	UK	8(9.6)

	Japan	12(14.4)
	Others and not reported	61(73.4)

USA: The United States of America; UK: The United Kingdom.

Supplementary Table S35. Demographic characteristics in the reports related to hemorrhage with edoxaban with prasugrel.

Variables		Values (n= 17)
Age group [n (%)]	<18 years	0
	≥18-<45 years	0
	≥45-<65 years	0
	≥65 years	14(82.3)
	Not reported	3(17.6)
Mean (SD) age (years)		74.07(4.6)
Median (range) age (years)		75(66-85)
Gender distribution [n (%)]	Male	11(64.7)
	Female	5(29.4)
	Unknown	1(5.8)
Year of receiving the report [n (%)]	2004-2008	0
	2009-2012	0
	2013-2016	4(23.5)
	2017-2020	7(41.1)
	2021-2024	6(35.2)
Top reporting countries [n (%)]	USA	4(23.5)
	Japan	13(76.4)

USA: The United States of America.

Supplementary Table S36. Demographic characteristics in the reports related to hemorrhage with edoxaban with ticagrelor.

**Variables**		**Values (n=3)**
Age group [n (%)]	≥45-<65 years	1(33.3)
	≥65 years	2(66.6)
Mean (SD) age (years)		74.6(14.6)
Median (range) age (years)		77(59-88)
Gender distribution [n (%)]	Male	3(100)
Year of receiving the report [n (%)]	2021-2024	3
Top reporting countries [n (%)]	Others and not reported	3(100)

Supplementary Table S37. Demographic characteristics in the reports related to hemorrhage with rivaroxaban with aspirin.

Variables		Values (n= 12801)
Age group [n (%)]	<18 years	3(0.02)
	≥18-<45 years	221(1.7)
	≥45-<65 years	2556(19.9)
	≥65 years	9268(72.4)
	Not reported	753(5.8)
Mean (SD) age (years)		71.52(11.02)
Median (range) age (years)		72(1-101)
Gender distribution [n (%)]	Male	7415(57.9)
	Female	5203(40.6)
	Unknown	270(2.1)
Year of receiving the report [n (%)]	2004-2008	0
	2009-2012	81(0.6)
	2013-2016	3940(30.77)
	2017-2020	8001(62.5)
	2021-2024	779(6.08)
Top reporting countries [n (%)]	USA	11572(90.39)
	Canada	167(1.3)
	UK	137(1.07)
	France	71(0.55)
	Japan	138(1.07)
	Others and not reported	716(5.5)

USA: The United States of America; UK: The United Kingdom.

Supplementary Table S38. Demographic characteristics in the reports related to hemorrhage with rivaroxaban with cilostazol.

**Variables**		**Values (n=89)**
Age group [n (%)]	<18 years	0
	≥18-<45 years	1(1.1)
	≥45-<65 years	13(14.6)
	≥65 years	63(70.7)
	Not reported	12(13.4)
Mean (SD) age (years)		74.48(11.54)
Median (range) age (years)		75(40-91)
Gender distribution [n (%)]	Male	62(69.6)
	Female	24(26.9)
	Unknown	3(3.3)
Year of receiving the report [n (%)]	2004-2008	0
	2009-2012	2(2.2)
	2013-2016	24(26.9)
	2017-2020	50(56.1)
	2021-2024	12(13.4)
Top reporting countries [n (%)]	USA	57(64)
	UK	1(1.1)
	Japan	20(22.4)
	Others and not reported	12(13.4)

USA: The United States of America; UK: The United Kingdom.

Supplementary Table S39. Demographic characteristics in the reports related to hemorrhage with rivaroxaban with clopidogrel.

**Variables**		**Values (n= 3431)**
Age group [n (%)]	<18 years	1(0.02)
	≥18-<45 years	46(1.34)
	≥45-<65 years	663(19.3)
	≥65 years	2322(67.9)
	Not reported	399(11.6)
Mean (SD) age (years)		71.4(10.85)
Median (range) age (years)		72(1-100)
Gender distribution [n (%)]	Male	2038(59.3)
	Female	1209(35.2)
	Unknown	178(5.1)
Year of receiving the report [n (%)]	2004-2008	0
	2009-2012	82(2.3)
	2013-2016	840(24.4)
	2017-2020	2072(60.3)
	2021-2024	437(12.7)
Top reporting countries [n (%)]	USA	2494(72.6)
	Canada	98(2.8)
	UK	144(4.1)
	France	67(1.9)
	Japan	74(2.1)
	Others and not reported	551(16)

USA: The United States of America; UK: The United Kingdom.

Supplementary Table S40. Demographic characteristics in the reports related to hemorrhage with rivaroxaban with dipyridamole.

Variables		Values (n= 36)
Age group [n (%)]	<18 years	0
	≥18-<45 years	1(2.7)
	≥45-<65 years	10(27)
	≥65 years	23(63.8)
	Not reported	2(5.5)
Mean (SD) age (years)		70.44(11.9)
Median (range) age (years)		72(44-92)
Gender distribution [n (%)]	Male	26(72.2)
	Female	9(25)
	Unknown	1(2.7)
Year of receiving the report [n (%)]	2004-2008	0
	2009-2012	0
	2013-2016	15(41.6)
	2017-2020	20(55.5)
	2021-2024	1(2.7)
Top reporting countries [n (%)]	USA	33(91.6)
	Japan	3(8.3)
	Others and not reported	

USA: The United States of America.

Supplementary Table S41. Demographic characteristics in the reports related to hemorrhage with rivaroxaban with prasugrel.

Variables		Values (n=107)
Age group [n (%)]	<18 years	0
	≥18-<45 years	6(5.6)
	≥45-<65 years	30(28)
	≥65 years	62(57.9)
	Not reported	9(8.4)
Mean (SD) age (years)		67.25(11.83)
Median (range) age (years)		68(36-90)
Gender distribution [n (%)]	Male	67(62.61)
	Female	37(34.5)
	Unknown	4(3.7)
Year of receiving the report [n (%)]	2004-2008	0
	2009-2012	0
	2013-2016	32(29.9)
	2017-2020	64(59.8)
	2021-2024	11(10.2)
Top reporting countries [n (%)]	USA	90(84.1)
	Japan	16(14.9)
	Others and not reported	1(0.9)

USA: The United States of America; UK: The United Kingdom.

Supplementary Table S42. Demographic characteristics in the reports related to hemorrhage with rivaroxaban with ticagrelor.

Variables		Values (n= 212)
Age group [n (%)]	<18 years	0
	≥18-<45 years	5(2.3)
	≥45-<65 years	53(25)
	≥65 years	120(56.6)
	Not reported	34(16.03)
Mean (SD) age (years)		68.1(10.8)
Median (range) age (years)		69(35-90)
Gender distribution [n (%)]	Male	124(58.4)
	Female	76(35.8)
	Unknown	11(5.1)
Year of receiving the report [n (%)]	2004-2008	0
	2009-2012	1(0.4)
	2013-2016	32(15)
	2017-2020	141(66.5)
	2021-2024	38(17.9)
Top reporting countries [n (%)]	USA	172(81.1)
	Canada	4(1.8)
	UK	6(2.8)
	Others and not reported	40(18.86)

USA: The United States of America; UK: The United Kingdom.

Supplementary Table S43. Demographic characteristics in the reports related to hemorrhage with warfarin with aspirin.

Variables		Values (n= 3841)
Age group [n (%)]	<18 years	102(2.6)
	≥18-<45 years	150(3.9)
	≥45-<65 years	747(19.4)
	≥65 years	2073(53.9)
	Not reported	768(19.9)
Mean (SD) age (years)		67.44(16.8)
Median (range) age (years)		71(0-97)
Gender distribution [n (%)]	Male	2085(54.2)
	Female	1329(34.6)
	Unknown	555(14.4)
Year of receiving the report [n (%)]	2004-2008	72(1.8)
	2009-2012	178(4.6)
	2013-2016	1171(30.48)
	2017-2020	1817(47.3)
	2021-2024	603(15.6)
Top reporting countries [n (%)]	USA	2213(57.6)
	Canada	138(3.5)
	UK	197(5.1)
	France	103(2.6)
	Japan	267(6.9)
	Others and not reported	923(24)

USA: The United States of America; UK: The United Kingdom.

Supplementary Table S44. Demographic characteristics in the reports related to hemorrhage with warfarin with cilostazol.

Variables		Values (n= 84)
Age group [n (%)]	<18 years	0
	≥18-<45 years	0
	≥45-<65 years	20(23.8)
	≥65 years	49(58.3)
	Not reported	15(17.8)
Mean (SD) age (years)		70.39(11.58)
Median (range) age (years)		74(42-90)
Gender distribution [n (%)]	Male	55(65.4)
	Female	24(28.5)
	Unknown	5(5.9)
Year of receiving the report [n (%)]	2004-2008	13(15.4)
	2009-2012	27(32.1)
	2013-2016	19(22.6)
	2017-2020	21(25)
	2021-2024	4(4.7)
Top reporting countries [n (%)]	USA	30(35.7)
	Japan	41(48.8)
	Others and not reported	13(15.4)

USA: The United States of America; UK: The United Kingdom.

Supplementary Table S45. Demographic characteristics in the reports related to hemorrhage with warfarin with clopidogrel.

Variables		Values (n= 2140)
Age group [n (%)]	<18 years	15(0.7)
	≥18-<45 years	44(2)
	≥45-<65 years	372(17.3)
	≥65 years	1267(59.2)
	Not reported	437(20.4)
Mean (SD) age (years)		70.25(12.93)
Median (range) age (years)		72(1-98)
Gender distribution [n (%)]	Male	1302(60.8)
	Female	717(33.5)
	Unknown	231(10.7)
Year of receiving the report [n (%)]	2004-2008	317(14.8)
	2009-2012	391(18.2)
	2013-2016	439(20.5)
	2017-2020	708(33)
	2021-2024	250(11.6)
Top reporting countries [n (%)]	USA	1267(59.2)
	Canada	53(2.4)
	UK	104(4.8)
	France	91(4.2)
	Japan	99(4.6)
	Others and not reported	526(24.5)

USA: The United States of America; UK: The United Kingdom.

Supplementary Table S46. Demographic characteristics in the reports related to hemorrhage with warfarin with dipyridamole.

Variables		Values (n= 155)
Age group [n (%)]	<18 years	19(12.2)
	≥18-<45 years	16(10.3)
	≥45-<65 years	33(21.2)
	≥65 years	59(38)
	Not reported	28(18)
Mean (SD) age (years)		54.78(24.6)
Median (range) age (years)		64(0-97)
Gender distribution [n (%)]	Male	95(61.2)
	Female	44(28.3)
	Unknown	26(16.7)
Year of receiving the report [n (%)]	2004-2008	33(21.2)
	2009-2012	33(21.2)
	2013-2016	19(12.2)
	2017-2020	59(38)
	2021-2024	11(7)
Top reporting countries [n (%)]	USA	70(45.1)
	UK	8(5.1)
	Japan	10(6.4)
	Others and not reported	67(43.2)

USA: The United States of America; UK: The United Kingdom.

Supplementary Table S47. Demographic characteristics in the reports related to hemorrhage with warfarin with prasugrel.

Variables		Values (n= 83)
Age group [n (%)]	<18 years	0
	≥18-<45 years	3(3.6)
	≥45-<65 years	26(31.3)
	≥65 years	35(42.1)
	Not reported	19(22.8)
Mean (SD) age (years)		64.2(13.7)
Median (range) age (years)		66(25-94)
Gender distribution [n (%)]	Male	60(72.2)
	Female	21(25.3)
	Unknown	2(2.4)
Year of receiving the report [n (%)]	2004-2008	0
	2009-2012	18(21.6)
	2013-2016	41(49.3)
	2017-2020	22(26.5)
	2021-2024	2(2.4)
Top reporting countries [n (%)]	USA	56(67.4)
	Japan	13(15.6)
	Others and not reported	14(16.8)

USA: The United States of America.

Supplementary Table S48. Demographic characteristics in the reports related to hemorrhage with warfarin with ticagrelor.

Variables		Values (n= 110)
Age group [n (%)]	<18 years	0
	≥18-<45 years	4(3.6)
	≥45-<65 years	9(8.1)
	≥65 years	68(61.8)
	Not reported	29(26.3)
Mean (SD) age (years)		68.6(10.89)
Median (range) age (years)		70(27-87)
Gender distribution [n (%)]	Male	65(59)
	Female	34(30.9)
	Unknown	11(10)
Year of receiving the report [n (%)]	2004-2008	0
	2009-2012	6(5.4)
	2013-2016	31(28.1)
	2017-2020	54(49)
	2021-2024	19(17.2)
Top reporting countries [n (%)]	USA	63(57.2)
	UK	9(8.1)
	Others and not reported	38(34.5)

USA: The United States of America; UK: The United Kingdom.
